# New insight into bacterial social communication in natural host: Evidence for interplay of heterogeneous and unison quorum response

**DOI:** 10.1371/journal.pgen.1008395

**Published:** 2019-09-17

**Authors:** Biswajit Samal, Subhadeep Chatterjee

**Affiliations:** 1 Lab of Plant-Microbe Interactions, Centre for DNA Fingerprinting and Diagnostics, Hyderabad, Telengana, India; 2 Graduate studies, Manipal Academy of Higher Education, Manipal, Karnataka, India; CNRS, FRANCE

## Abstract

Many microbes exhibit quorum sensing (QS) to cooperate, share and perform a social task in unison. Recent studies have shown the emergence of reversible phenotypic heterogeneity in the QS-responding pathogenic microbial population under laboratory conditions as a possible bet-hedging survival strategy. However, very little is known about the dynamics of QS-response and the nature of phenotypic heterogeneity in an actual host-pathogen interaction environment. Here, we investigated the dynamics of QS-response of a Gram-negative phytopathogen *Xanthomonas* pv. *campestris* (*Xcc*) inside its natural host cabbage, that communicate through a fatty acid signal molecule called DSF (diffusible signal factor) for coordination of several social traits including virulence functions. In this study, we engineered a novel DSF responsive whole-cell QS dual-bioreporter to measure the DSF mediated QS-response in *Xcc* at the single cell level inside its natural host plant *in vivo*. Employing the dual-bioreporter strain of *Xcc*, we show that QS non-responsive cells coexist with responsive cells in microcolonies at the early stage of the disease; whereas in the late stages, the QS-response is more homogeneous as the QS non-responders exhibit reduced fitness and are out competed by the wild-type. Furthermore, using the wild-type *Xcc* and its QS mutants in single and mixed infection studies, we show that QS mutants get benefit to some extend at the early stage of disease and contribute to localized colonization. However, the QS-responding cells contribute to spread along xylem vessel. These results contrast with the earlier studies describing that expected cross-induction and cooperative sharing at high cell density *in vivo* may lead to synchronize QS-response. Our findings suggest that the transition from heterogeneity to homogeneity in QS-response within a bacterial population contributes to its overall virulence efficiency to cause disease in the host plant under natural environment.

## Author summary

Pathogenic bacteria synchronize and coordinate the production of virulence associated function-components in a density dependent fashion via quorum sensing. In general, QS-response and regulation has been studied under laboratory conditions *in vitro*, where the QS-responding bacterial population exhibits heterogeneous QS-response with the emergence of both QS responders and non-responders irrespective of their parental kind, as a possible bet hedging strategy. However, very little is known about the dynamics of QS-response inside the host. Using *Xanthomonas campestris* pv. *campestris* (*Xcc*) and cabbage as a model plant pathogen-host, we show that there is stage specific interplay of heterogeneous and homogeneous QS-response in the wild-type *Xcc* population inside the host plant. We show that at the initial stage of the disease, *Xcc* maintains a stochastically heterogeneous population wherein, the QS non-responders are localized locally and QS-responders contribute to the migration and spread. However at the later stage of disease, the non-responders are outcompeted by the responders, thus minimizing QS signal benefit and in turn maximizing the utilization and optimizing limited recourses in the host. Our findings suggest that the interplay of heterogeneity and homogeneity in QS-response gives a stage specific adaptive advantage in a host-pathogen natural environment.

## Introduction

Pathogenic bacteria coordinate several social behaviors via production, secretion and perception of diverse diffusible cell-cell signaling molecules by a process called quorum sensing (QS). QS synchronizes the bacteria to perform social task in unison by coordinating production of exo-products as ‘public goods’ that are beneficial to the population as a whole. Such social tasks include the production of virulence associated function-components involved in biofilm formation, extracellular enzymes, extracellular polysaccharide and surfactants that promote motility and spread [1, 2, 3, 4]. The simplified model is that QS coordinates the collective bacterial behavior in unison to maximize the inclusive fitness of individual cells in the community at high cell density, thus avoiding costly production of public goods at low cell density [5, 6, 7].

Although QS has been associated with cooperation at high cell density, recent experimental and theoretical modelling studies in pathogenic bacteria such as *Pseudomonas*, *Vibrio* and *Xanthomonas* have demonstrated that QS-response is complex; as bacteria exhibit reversible non-genetic phenotypic heterogeneity in QS-response generating two distinct sub-populations of QS-responsive and non-responsive cells under artificial laboratory conditions [8, 9, 10, 11]. It has been argued that generation of non-genetic phenotypic heterogeneity in bacterial QS-response by stochastic fate determination in an isogenic population may be a bet-hedging survival strategy that enables the population adaptation to fluctuating environmental condition [9]. In other words, heterogeneity in performing social task may have adaptive functions, such as division of labour and sharing of environmental resources.

It has been proposed that heterogeneity in QS may arise due to highly sensitive QS-response, which may result in intrinsic stochasticity of QS-response at lower concentrations of auto-inducer [12]; production of sub-optimum level of QS signals even at high cell density [8]; or inherent heterogeneity in QS-response even in the presence of saturating concentration of QS signals [9]. Recent theoretical modelling study suggested that coupling mediated by quorum sensing between ecological and population dynamics can induce phenotypic heterogeneity in a QS experiencing microbial population [11]. Although heterogeneity in QS-response has been studied under artificial laboratory conditions in bacterial pathogens, very little is known about the QS-response dynamics in natural environment particularly inside the host. This raises interesting and significant question regarding the bacterial behavior towards QS heterogeneity for better adaptation in natural host, under fluctuating environmental condition as well as change in cell density.

To address the above question, here we investigated whether pathogenic bacteria exhibit QS heterogeneity and its possible role towards social-cooperation and adaptation within its natural plant host, using plant pathogen *Xanthomonas campestris* pv. *campestris* (*Xcc*) as a model system that causes black rot disease of cabbage and several other cruciferous plants [13]. In *Xanthomonas* group of phytopathogens, QS is mediated by the synthesis and perception of fatty acid signaling molecules called DSF (Diffusible Signal Factor; *cis*-11-methyl-2-dodecenoic acid) and its derivatives, which are involved in regulation of expression of several virulence-associated factors such as motility, biofilm formation, production of extracellular polysaccharides (EPS) and extracellular enzymes [14, 15, 16, 17]. The phytopathogen *Xcc* is able to detect its population density through QS via production (via RpfF; DSF synthase) and perception (via RpfC; DSF sensor) of DSF as quorum signal; where the bacteria significantly depend on QS regulation to coordinate its colonization and infection of plant hosts [2, 18].

In this study, we visualized the spatial and temporal dynamics of DSF dependent QS-response at the single cell level in the wild-type *Xcc* during the early and late stage of the disease progression in the host cabbage plant. We have shown that at early stage of disease, the QS non-responsive cells coexist with QS-responsive cells in the wild-type population. However, at the late stage of the disease, the QS-response was more homogeneous. Using single and mixed infection studies *in planta* with various bioreporter strains of the wild-type *Xcc* 8004 (QS performers/responders; that are able to synthesize and sense the DSF to perform QS) and its QS deficient mutants(QS non-performers/non-responders/cheaters; that are defective in either synthesis or sensing of DSF and hence unable to perform QS, including QS null Δ*rpfF* and QS blind Δ*rpfC*), we have shown that inspite of *in planta* survival of cheaters in the QS-induced *Xcc* population at the early stages of disease, the QS non-responders (i.e. QS cheaters) are outcompeted by the wild-type QS responders at the later stage of disease, as there is significant decline in growth, migration and spread of non-responders, resulting in a more homogeneous QS-response within the quorum induced bacterial population. These results contrast with the earlier studies; those describe that expected cross-induction and cooperative sharing of public goods at high cell density *in vivo* may lead to synchronize QS-response [4, 7, 10, 18, 19, 20]. Here, we argue that under natural condition during bacterial colonization of host plants, the interplay between heterogeneity and homogeneity towards QS-response may provide a stage specific adaptive advantage to the bacterial populations.

## Results

### DSF responsive *in vitro* QS heterogeneity is temporal and bi-modally distributed in *Xcc* population

Previously we have demonstrated that the wild-type *Xcc* exhibits heterogeneity in the DSF mediated QS-response *in vitro* which is a reversible stochastic phenomenon [9]. However, to investigate the detailed dynamics of DSF mediated QS-response in *Xcc in vitro* as well as more importantly inside the host plant at the single cell level, we engineered a DSF responsive whole-cell QS dual-bioreporter that harbours a gene encoding monomeric red fluorescent protein fused to the DSF responsive promoter *eng* (*P*_*eng*_:*rfp*) to monitor the QS-response and a constitutive *P*_*kan*_:*gfp* marker gene to enable an accounting for all the bioreporter cells in the isogenic bacterial population both *in vitro* and *in vivo*. The above *Xcc* dual-biosensor expressed red fluorescence in response to DSF, and green fluorescence constitutively whose intensity was independent of the amount of DSF produced by it (S1 Fig).

To analyze the detailed QS induction dynamics under artificial laboratory conditions, we performed both *in vitro* confocal laser-scanning microscopy (CLSM) and colony forming unit (CFU) studies using the DSF responsive whole-cell dual-bioreporter of the wild-type *Xcc* 8004 in the nutrient rich PS medium, along with its DSF deficient Δ*rpfF* mutant harbouring the dual-bioreporter construct (p*P*_*kan*_:*gfp*-*P*_*eng*_:*rfp*) as a QS negative control alone and in the presence of external DSF at optimal level (i.e. 4.84 μM) separately (*see* Materials and Methods). At each time period, all the *gfp* expressing cells were considered for bacterial cell density calculation, but the cells expressing both *gfp* and *rfp* were considered for QS induction calculation. The quorum induced average RFP pixel intensity per bacterial cell was represented in arbitrary units (A.U.). The average cell-normalized RFP fluorescence of “*Xcc* 8004 (p*P*_*kan*_:*gfp*-*P*_*eng*_:*rfp*)” as well as “*Xcc* Δ*rpfF* (p*P*_*kan*_:*gfp*-*P*_*eng*_:*rfp*) supplemented with external DSF” increased in a typical density-dependent fashion 12 hr onwards from a initial culture density of ~ 6 × 10^4^ cells ml^-1^, with maximum induction (average RFP pixel intensity ~ 44 A.U.) occurring between 20–28 hrs of inoculation with approximately 10^8^ to 10^9^ cells ml^-1^ culture. Analysis of the fractions of induced (average RFP pixel intensity > 9 A.U.) and uninduced (average RFP pixel intensity < 7 A.U.) cells of *Xcc* 8004 (p*P*_*kan*_:*gfp*-*P*_*eng*_:*rfp*) and “*Xcc* Δ*rpfF* (p*P*_*kan*_:*gfp*-*P*_*eng*_:*rfp*) supplemented with external DSF” revealed that the percentage of QS-induced (RFP^+^) cells increased with time, where only ~ 80–85% cells in the population exhibited QS-induced state even at high cell density (~ 10^9^ to 10^10^ cells ml^-1^) between 24 to 44 hr of growth (S2 Fig, S3 Fig). However, at any sampling point on 20 hrs onward only, the *Xcc* Δ*rpfF* (p*P*_*kan*_:*gfp*-*P*_*eng*_:*rfp*) cells were able to exhibit little RFP fluorescence with average pixel intensity of ~ 3 to 6 A.U. towards minimal promoter activity within population (S4 Fig). These results revealed that *Xcc* experiences a temporal QS heterogeneity in response to DSF at high cell density *in vitro*.

In parallel, the analysis of distribution of constitutive GFP and DSF responsive RFP fluorescence intensity of at least 100 individual cells for each strain at mentioned sampling times by confocal microscopy revealed the co-existence of both QS-induced and QS uninduced sub-populations with bimodal QS-distribution in the quorum induced populations of *Xcc* 8004 (p*P*_*kan*_:*gfp*-*P*_*eng*_:*rfp*) at 24 hr and 36 hr of growth (S5A Fig). With no substantial QS-response in the DSF deficient *Xcc* Δ*rpfF* (p*P*_*kan*_:*gfp*-*P*_*eng*_:*rfp*) population even at sufficiently high cell density (S5B Fig), the bimodal QS-distribution pattern could be restored under similar conditions of growth in the population of *Xcc* Δ*rpfF* (p*P*_*kan*_:*gfp*-*P*_*eng*_:*rfp*) upon initial supplementation with 4.84 μM external DSF into the culture (S5C Fig). However, the maximum bimodal gene expression was observed with strongest QS-response (average RFP pixel intensity ~ 47 A.U.) within the QS-induced bacterial population of “wild-type *Xcc*” as well as “its DSF deficient *Xcc* Δ*rpfF* supplemented with DSF” at 24 hrs of growth, but not in the QS null *Xcc* Δ*rpfF* population alone (S5D Fig).

### The *in planta* colonization of *Xcc* is spatio-temporally regulated within its natural host

*Xcc* causes black rot disease of cruciferous plants such as cabbage, cauliflower [13]; where it gains entry inside water conducting xylem vessels of the host plant through natural openings at the tip of the leaf known as hydathodes or through leaf wounds [21, 22]. During *in planta* colonization, *Xcc* group of phytopathogens primarily localize and grow within the vascular regions, and subsequently can escape to surrounding mesophyll regions at the late stage of the infection [23, 24].

To analyze the detailed *in planta* localization and growth of *Xcc* during disease progression, we performed wound infection assays in cabbage leaves with the wild-type *Xcc* harbouring the DSF responsive dual-bioreporter construct and visualized the *in planta* distribution patterns for the bacterial population by confocal microscopy. The growth of the wild-type *Xcc* 8004 harbouring the dual-bioreporter construct (p*P*_*kan*_:*gfp*-*P*_*eng*_:*rfp*) was similar to the wild-type *Xcc* 8004 strain alone (S6A Fig). Although, the bacterial localization was found to be comparatively higher within the proximal vascular regions compared to their surrounding mesophyll regions up to 12 dpi, the *Xcc* 8004 (p*P*_*kan*_:*gfp*-*P*_*eng*_:*rfp*) population size increased significantly faster in the proximal xylem vessel lumens from the initial population size of 10^4^ cells to 10^5^ cells per leaf within 3 days of incubation as compared to their surrounding mesophyll regions (S6B Fig).

### *Xcc* experiences both heterogeneous and homogeneous QS-responses during early and late stages of disease progression respectively under hostile environment *in planta*

We analyzed the DSF dependent QS-response of *Xcc* population at single cell level or in cell-aggregates inside the host plant by visualizing the initiation of QS-induced *rfp* expression patterns for wild-type *Xcc* dual-biosensor cells spanning both vascular and mesophyll regions of the wound inoculated cabbage leaves by confocal microscopy.

**Fig 1:**
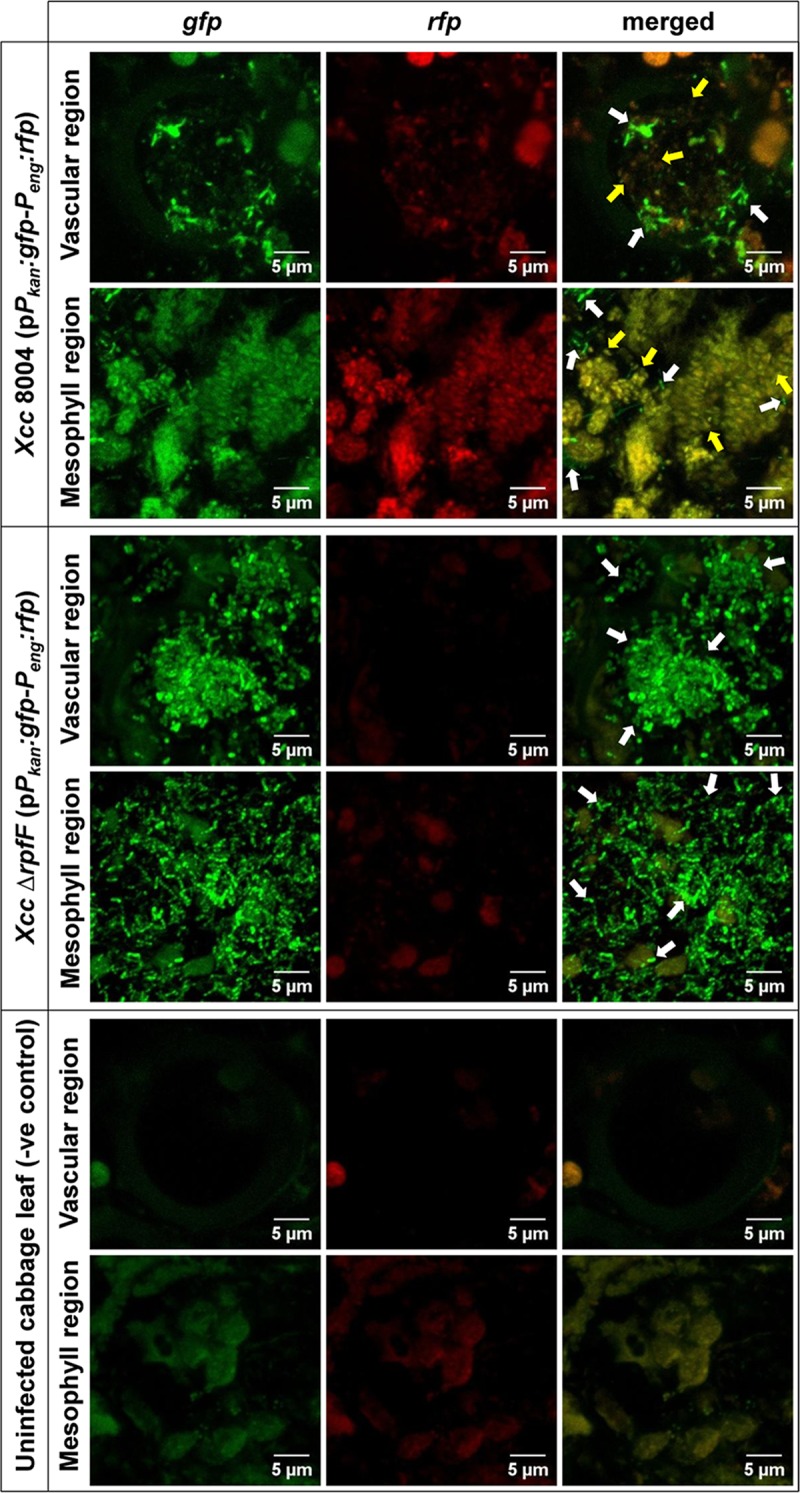
*Xcc* experiences DSF responsive QS heterogeneity during early stage of disease establishment within host plant. 40 days old healthy cabbage leaves were clipped with low cell density cultures (~ 10^6^ cells ml^-1^) of the wild-type *Xcc*-biosensor strain and the inoculated leaves were scanned under CLSM for the QS-induced *rfp* expression patterns for all constitutive *gfp* expressing dual-bioreporter cells spanning its proximal non-symptomatic green vascular and mesophyll regions (within 1 cm distance from beyond the diseased symptom periphery) at regular intervals upto 12 dpi. In parallel, its DSF synthesis mutant Δ*rpfF*-biosensor strain was used as a QS negative control strain. The uninfected cabbage leaves were used as control plant to visualise plant autofluorescence under CLSM at same exposure. Shown in the above figure are the representative CLSM pictures depicting the heterogeneous QS-response within wild-type *Xcc* 8004 dual-bioreporter populations spanning both vascular and mesophyll regions of infected cabbage leaves on 6 dpi, along with *Xcc* Δ*rpfF* (as QS negative control) and uninfected cabbage leaves (as a control plant). The panels from left to right show *gfp*, *rfp* and their merged images respectively. For each bioreporter strain, top and bottom panels represent the bacterial populations localizing the transverse sections of individual xylem vessels and mesophyll regions respectively. Yellow arrows; QS-induced cells, White arrows; QS uninduced cells. For uninfected cabbage leaves (i.e. control plant), top and bottom panels represent the plant autofluorescence (without any bacterial populations) for transverse sections of individual xylem vessels and mesophyll regions respectively. Images were prepared using FIJI (image J) software. Scale bars on each panel, 5 μm.

*In planta*, both the bacterial populations exhibited no detectable red fluorescence on leaves initially after inoculation (0 day post inoculation; 0 dpi), but could be readily detected because of their bright green fluorescence. However, heterogeneously QS-induced populations with sufficient amount of QS dependant red fluorophore expression were observed for only wild-type *Xcc* 8004 dual-bioreporter within both proximal vascular and mesophyll leaf regions on 6 dpi (i.e. the last dpi before the appearance of characteristics diseased phenotype on the inoculated leaves, considered as “early stage of disease establishment”), but not for DSF synthesis mutant *Xcc* Δ*rpfF* dual-bioreporter under similar conditions except the background plant autofluorescence of uninfected control cabbage leaves (Fig 1). These results revealed that *Xcc* experience the QS heterogeneity in response to DSF at single cell level during early stage of disease establishment within its natural host plant.

**Fig 2:**
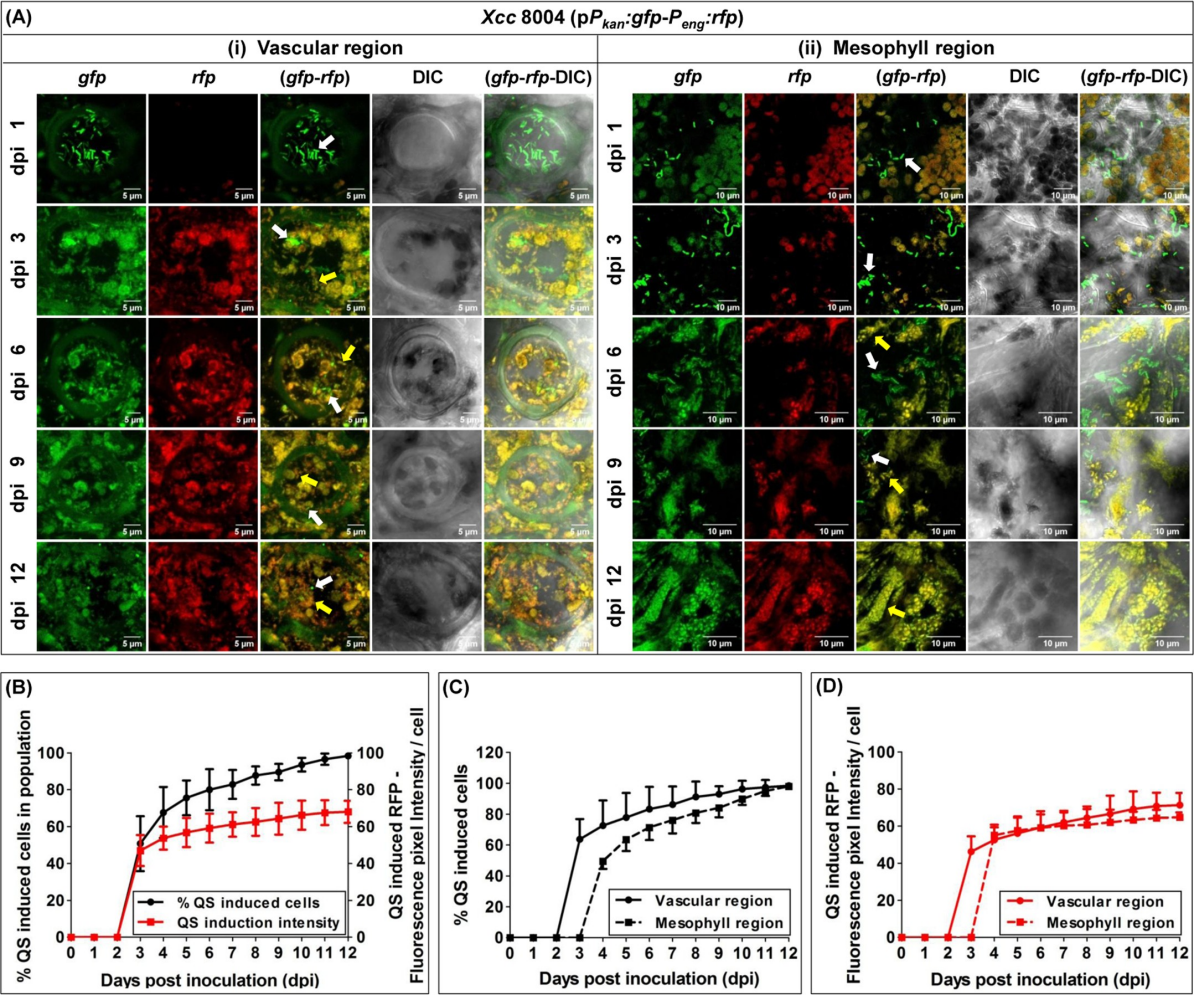
With spatio-temporal distribution, *Xcc* experiences QS homogeneity during late stage of disease establishment *in planta*. **(A)** Representative CLSM pictures showing the differential spatio-temporal localization and QS distribution dynamics for the wild-type *Xcc* 8004 dual-bioreporter populations within both xylem vessels and their surrounding mesophyll regions of the infected cabbage leaves upto dpi 12. **(i)** Transverse sections of individual xylem vessels. Scale bars on each panel, 5 μm. **(ii)** Mesophyll regions. Scale bars on each panel, 10 μm. The panels from top to bottom represent the individual and merged images of green and red fluorescence and bright field for dpi 1, 3, 6, 9 and 12. The panels for each dpi (left to right) show *gfp*, *rfp*, *gfp*-*rfp* merged, DIC, *gfp*-*rfp*-DIC merged images respectively. Yellow arrows; QS-induced cells, White arrows; QS uninduced cells. Images were prepared using ImageJ-win32 software. **(B)** Quantifications of total QS-induced bacterial population with QS intensity per bacterial cell within *Xcc* 8004 bioreporter population *in planta*. **(C)** Percentage QS-induced *Xcc* 8004 bioreporter populations, and **(D)** QS intensity per *Xcc* 8004 bioreporter cells within the populations; localized within vascular and mesophyll regions. Bacterial no. and QS-induced fluorescence pixel intensities were calculated using FIJI (image J) and ZEN softwares. Data analysis was done by taking six different confocal images as samples for each strain at a time with the experimental repeat of at least thrice and represented with Mean ± SD.

The detailed confocal microscopic analysis revealed the spatio-temporally regulated QS initiation and distribution within the wild-type *Xcc* 8004 dual-bioreporter population *in planta* within the infected cabbage leaves upto dpi 12; spanning both proximal vascular and their surrounding mesophyll area respectively (Fig 2A). The QS dependant expression of red fluorophore was only visible in the wild-type *Xcc* 8004 dual-bioreporter population from day 3 onwards post inoculation only, where almost half of the population (~ 50.75%) was found to be QS-induced producing sufficient amount of red fluorophore (with average fluorescence pixel intensity of ~ 47 A.U.) to be detected other than background red fluorescence due to plant chloroplasts and other plant debris. By the 6 days post inoculation, approximately 80% of the *Xcc* population was found to be QS-induced with higher quorum intensity per cell (average red fluorescence pixel intensity ~ 59 A.U.). However, interestingly at the later stage at dpi 12, almost all cells of the *Xcc* population were found to be QS-induced (≥ 98%) with much stronger QS induction per cell (average red fluorescence pixel intensity ~ 68 A.U.) compared to the *in vitro* QS-response in culture (Fig 2B). Further study to understand the spatial distribution of Quorum response along with *Xcc* localization in both proximal vascular and its surrounding mesophyll regions indicated a much earlier Quorum response within xylem vessels with approximately 65% induced population on 3 dpi as compared to a late Quorum response within surrounding mesophyll extracellular regions with approximately 45% induced population 4 dpi. *Xcc* maintained a significantly larger quorum size within vascular regions compared to mesophyll regions with maximum heterogeneity in QS-response during early stage of disease progression upto 6 days post inoculation. However, more homogeneously QS-induced populations with more than 98% quorum induced cells were observed during late stage of disease progression from 6 dpi onwards in both the regions (Fig 2C and 2D).

**Fig 3:**
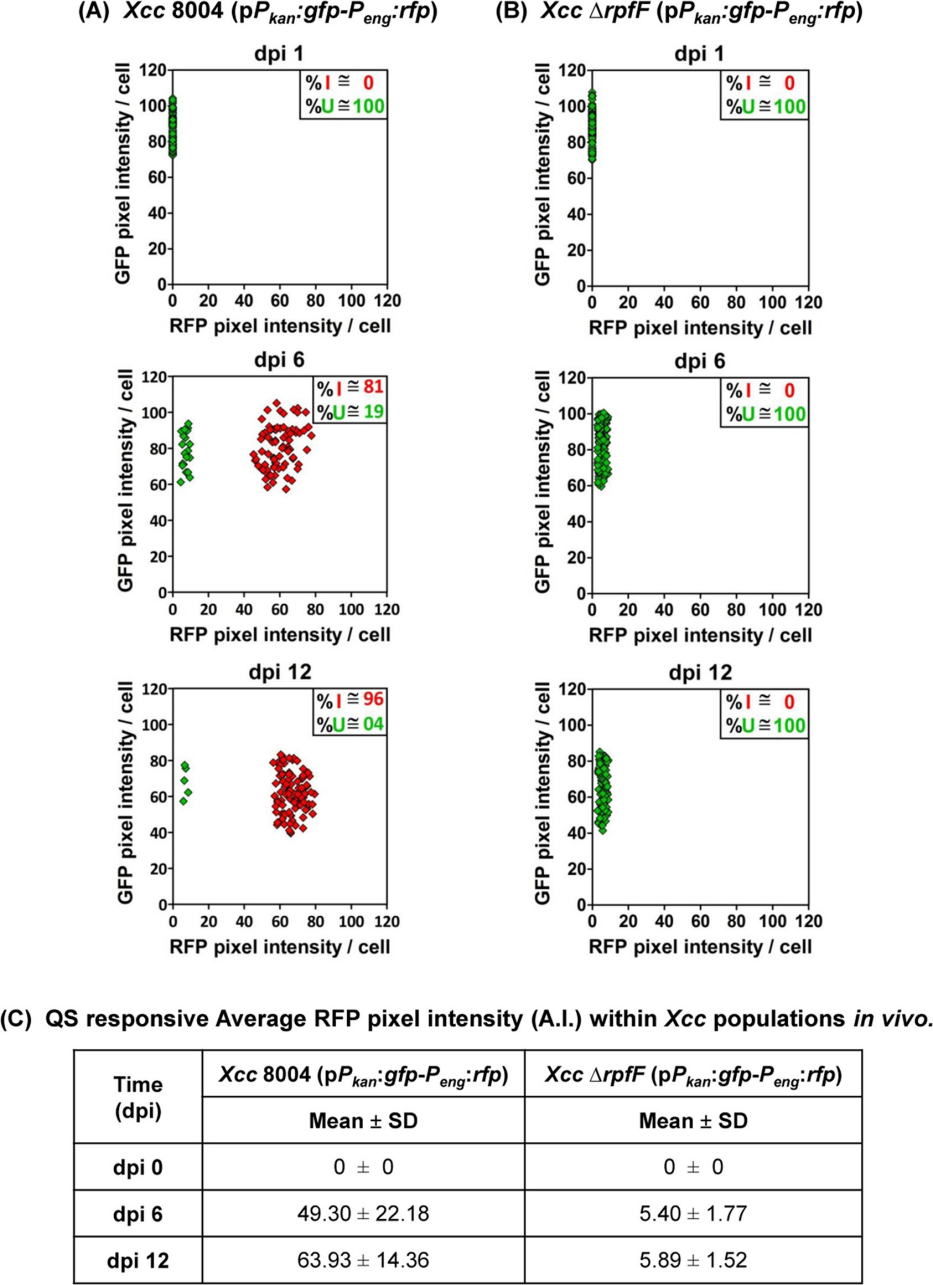
*Xcc* exhibits a typical bi-modal QS-response during early stage of disease establishment *in planta*. CLSM analysis of 100 representative bacterial cells for their constitutive *gfp* and QS-responsive *rfp* expression patterns within the dual-bioreporter populations of **(A)** wild-type *Xcc*, and **(B)** *Xcc* Δ*rpfF* (as a QS negative control) at different stages of growth *in planta*. The panels for each strain (top to bottom) represent QS distribution within the population on dpi 1 (i.e. initially after inoculation), dpi 6 (i.e. early stage of disease establishment) and dpi 12 (i.e. late stage of disease establishment) respectively. Each diamond symbol represents a single bacterial bioreporter cell observed under CLSM. Red diamonds; QS-induced bioreporter cells (cells expressing both *gfp* and *rfp*), Green diamonds; QS uninduced bioreporter cells (cells expressing only *gfp*). % I; Percent QS-induced population, % U; Percent QS uninduced population. **(C)** Quantification of the average QS-responsive RFP pixel Intensity (A.I.) within the bacterial populations during different stages of infection *in vivo*. On each dpi, data analysis was performed (using ZEN software) by taking four different confocal images as samples for each strain at a time with the experimental repeat of at least thrice and represented as Mean ± SD; where, both GFP and RFP fluorescence pixel intensities were represented in Arbitrary Units (A.U.).

In parallel, the confocal microscopy analysis towards distribution of constitutive GFP and DSF responsive RFP fluorescence intensity for at least 100 representative individual cells from each strain on mentioned sampling days {i.e. dpi(s) 1, 6 and 12} revealed the co-existence of both QS-induced and QS uninduced sub-populations with a typical and strong bimodal QS-distribution (in ~ 81:19 ratio) in the quorum induced heterogeneous population of wild-type *Xcc* 8004 (p*P*_*kan*_:*gfp*-*P*_*eng*_:*rfp*) on dpi 6 at early stage of disease establishment, but a comparatively weaker bimodal QS-distribution within the strongly quorum induced population dominated largely with QS responders over QS non-responders (in ~ 96:04 ratio) on dpi 12 at late stage of disease establishment (Fig 3A and 3B). During maximum bimodal gene expression in the heterogeneously QS-induced *Xcc* population on dpi 6, the bacteria exhibited a strong QS-response (average RFP pixel intensity ~ 49 A.U.) similar to *in vitro*. However, the average QS intensity was comparatively stronger (average RFP pixel intensity ~ 64 A.U.) with a weaker bimodal gene expression in the wild-type *Xcc* population on dpi 12, unlike *in vitro*. As a QS negative control, the DSF deficient QS null *Xcc* Δ*rpfF* population was unable to exhibit any QS-response and bimodal gene expression alone in the plant host upto dpi 12 (Fig 3C).

In addition to the QS induction and localization studies with the wild-type *Xc*c dual-bioreporter strain, we also observed the role of DSF dependent QS-response towards spatio-temporal bacterial localization *in planta* using two other previously reported biosensor strains; β-glucuronidase (GUS) assay with DSF dependent QS-responsive GUS reporter strain of wild-type *Xcc* 8004 {*Xcc* 8004 (pLAFR/*P*_*eng*_:*gusA*)} and confocal microscopy with the wild-type *Xcc* harbouring a DSF responsive *gfp* reporter {*Xcc* 8004(pKLN55/*P*_*eng*_:*gfp*)}. Analysis of GUS and GFP expression pattern *in planta* further corroborated the heterogeneity in the DSF dependent QS-response and the pattern of bacterial colonization; wherein, the *Xcc* cells colonizing the xylem vessels exhibited initiation of QS induction followed by the escape of QS-induced *Xcc* cells to the surrounding mesophyll region (S7 Fig). Our *in planta* GUS study gives a clear idea that QS induction happens to be initiate within vascular region followed by the distribution of the QS-induced bacterial population spanning both vascular and their surrounding mesophyll regions at different stages of infection.

**Fig 4:**
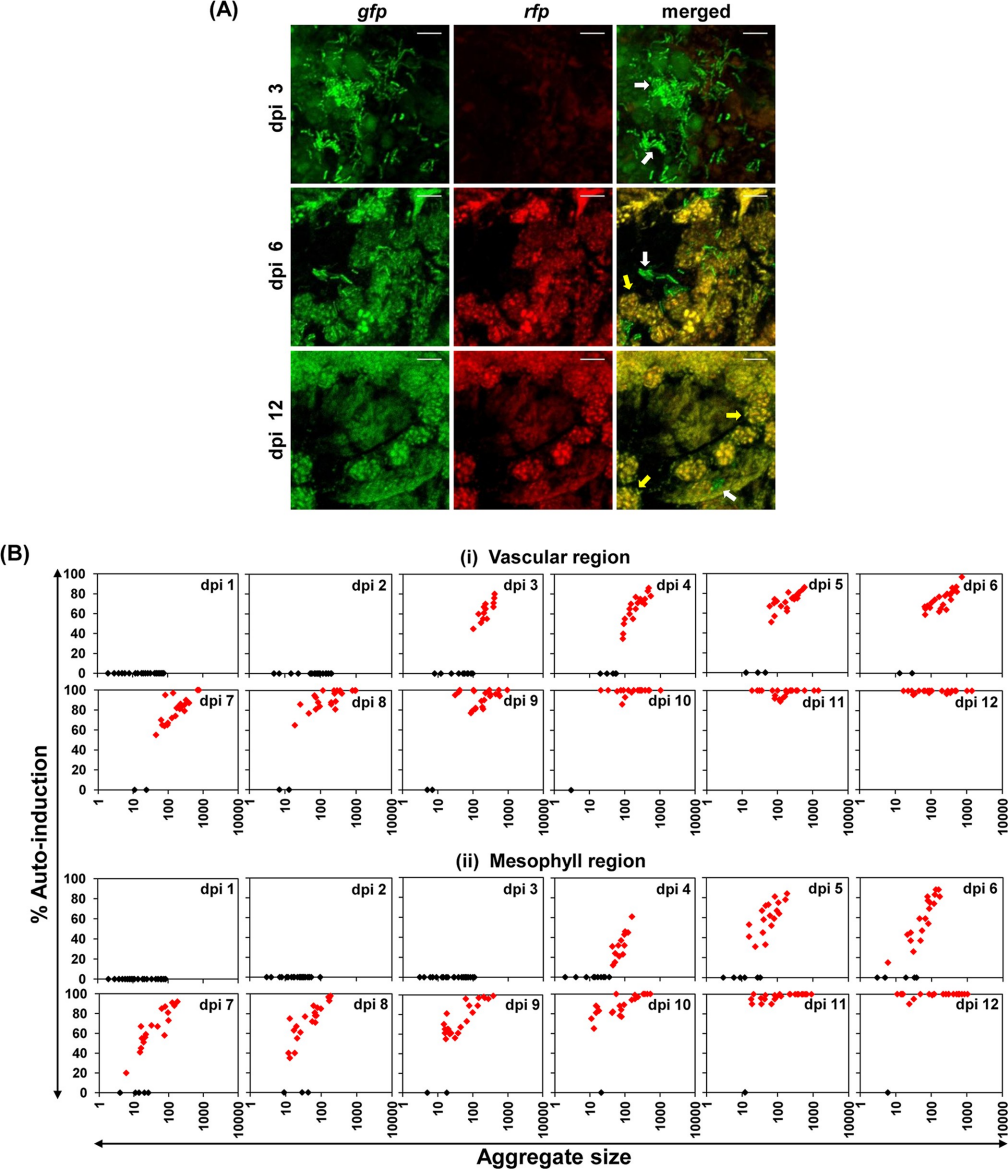
Individual *Xcc* aggregates within host plant also display spatio-temporal regulation towards QS heterogeneity *in planta*. **(A)** The representative CLSM pictures depicting the heterogeneous and homogeneous QS-response within individual bacterial aggregates of *Xcc* 8004 dual-bioreporter cells *in planta* at different stages of disease. The panels from top to bottom show dual-bioreporter cells spanning transverse sections of leaf mesophyll regions on dpi 1, 6 and 12 respectively. For each sampling dip, the panels from left to right show *gfp*, *rfp* and their merged images respectively. Only *gfp* expressing cells; QS uninduced cells (white arrows), Both *gfp* and *rfp* expressing cells; QS-induced cells (yellow arrows). Images were prepared using FIJI (image J) software. Scale bars on each panel, 5 μm. **(B)** Spatio-temporal QS induction and distribution dynamics within different sized bacterial aggregates *in planta* spanning both vascular (upper panels) and mesophyll (lower panels) regions upto dpi 12, indicating heterogeneous QS-response within the representative individual quorum induced bacterial aggregates without any spatial restriction towards QS distribution. On each sampling dpi, at least 24 aggregates from six infected leaves were randomly analyzed to determine the aggregate size and percentage QS induction with in the aggregate. The aggregate size was estimated from the volume of each aggregate divided by volume of each bacterial cell. Each diamond symbol represents a given cell aggregate observed under CLSM. Black diamonds; Uninduced aggregates, Red diamonds; Induced aggregates.

To understand the distribution of QS-induced and uninduced cells within the bacterial aggregates or microcolonies of different size inside the area of colonization, we analyzed the percent of QS induction within different bacterial aggregates spanning both vascular as well as the surrounding mesophyll region by taking 24 different representative *Xcc* dual-bioreporter aggregates at different time points post infection. Initially, even cell aggregates as large as 100 cells did not exhibit QS induction till 2 dpi within vascular regions and upto 3 dpi within mesophyll regions, as measured by red fluorescence indicative of DSF dependent QS induction. The inductions of QS-response in individual cells in the aggregates were more evident in the vascular and mesophyll region after 2 and 4 dpi, respectively. The percent of QS induction was much higher in similar sized bacterial aggregates within vascular region compared to the surrounding mesophyll area. Relatively, smaller aggregates were able to get auto-induced within vascular regions from 3 dpi onwards. The cell aggregate size at which QS induction was observed was progressively smaller. A more heterogeneously distributed quorum response was observed within almost similar aggregate sizes from 3 to 8 dpi in the vascular region and from 4 to 10 dpi in the mesophyll region, which thereafter became more homogeneous gradually upto 12 dpi with almost 100% induction in larger aggregates at a given dpi (Fig 4).

### A declined fitness of QS non-responders in growth, migration and cell-aggregate formation inclusively leads to a more homogeneously QS-induced *Xcc* population during the late stage of disease establishment

Despite several earlier reports describing high fitness of Gram-negative QS non-responders in animal pathogens [25, 26, 26], some recent reports argue that the spatial structure, occurrence of well separated microcolonies of wild-type and QS non-responders in early-stage infections *in vivo* may limit sharing of public goods by QS null mutants which may limit mutant fitness [19, 27]. However, in our QS induction and localization studies in the wild-type *Xcc* indicated no spatial structures that could limit sharing of public goods, as both QS-induced (responsive) and uninduced (non-responsive) cells were localized together in similar size cell aggregates or microcolonies. We therefore wanted to address whether the QS non-responders have fitness disadvantage at late stage of infection or there is excess sharing of QS signal within the population which could results in a homogeneous QS-responding population at the late stage of disease establishment in host plant.

We performed *in planta* competition assays with individual and mixed (in 1:1 ratios) inoculums separately using different constitutive reporter cells of wild-type *Xcc* 8004 (i.e. QS responders; able to produce and sense DSF), *Xcc* Δ*rpfF* (i.e. QS null mutant; defective in DSF synthesis but able to sense DSF) and *Xcc* Δ*rpfC* (i.e. QS blind mutant; hyper-producer of DSF but defective in DSF sensing) harbouring either a constitutive *gfp* (or) *mCherry* marker gene, to elucidate the QS-response benefits towards survival fitness among QS^+^ versus QS^-^ cells of *Xcc* population at different cell densities within host plant (*see* Materials and Methods); where the bioreporter cells were observed under a CLSM along with *in planta* CFU assay to analyse the growth/localization, migration, survibility and cell-aggregate formation patterns for each bacterial population within the host plant during different stages of disease establishment.

The preliminary CLSM analysis for *in planta* competition assay with single and co-cultures of wild-type *Xcc* 8004 bioreporters expressing either constitutive *P*_*kan*_:*gfp* or *P*_*lac*_:*mCherry* ruled out any significant fitness difference due to different constitutive marker genes under differential promoter activities upto dpi 12, indicating the similar survival fitness of both the reporter strains within 1 cm^2^ proximal green regions of infected cabbage leaves inspite of harbouring different reporter constructs (S8 Fig).

**Fig 5:**
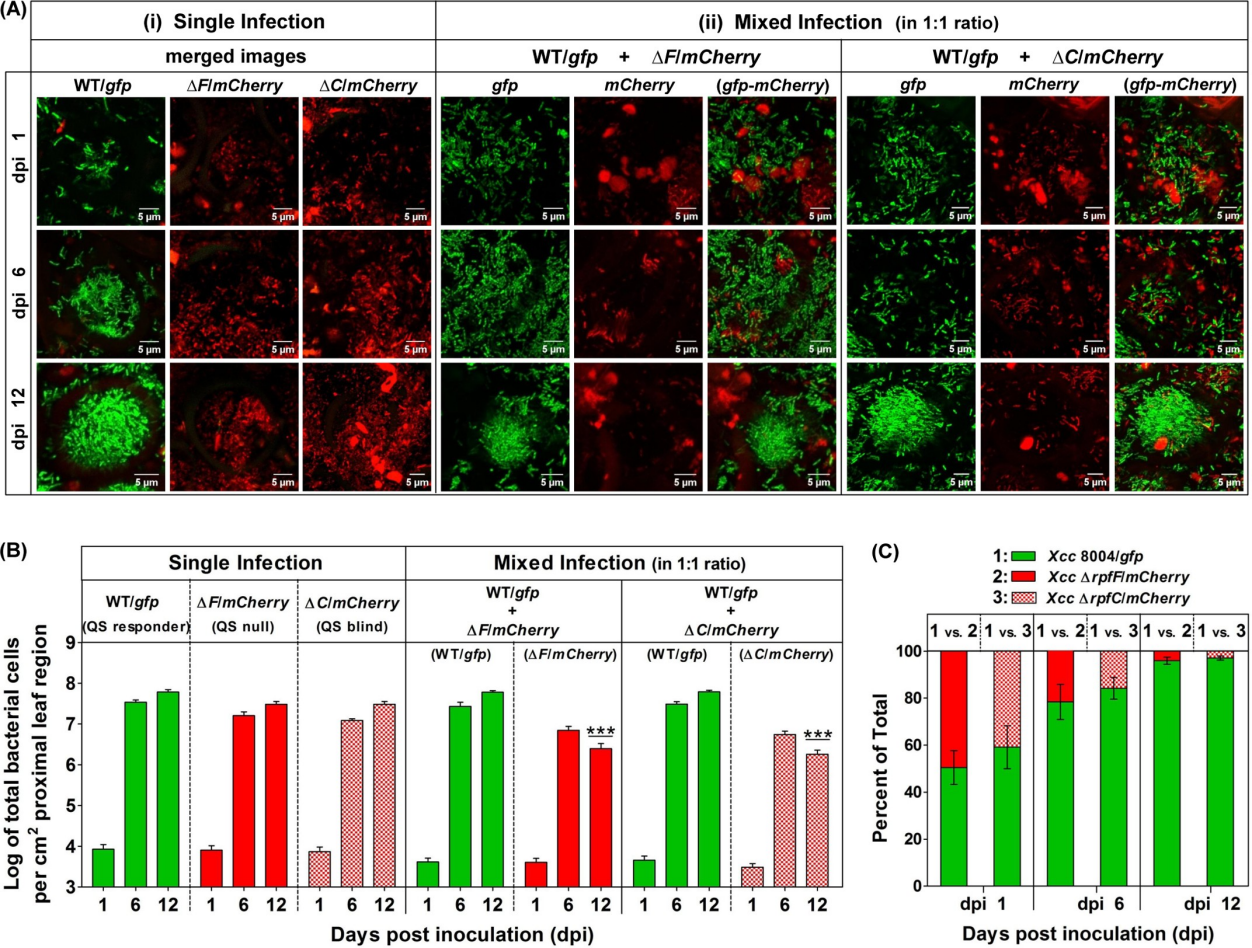
QS responders restrict the fitness of QS mutants in *Xcc* during late stage of infection. *In planta* competition assay indicating a significantly compromised survival fitness of QS mutants (QS^-^) in presence of QS responders (QS^+^) of *Xcc* at late stage of infection, where **(A)** Representative CLSM pictures for **(i)** Single infection (with the panels of merged images of green and red fluorescence), and **(ii)** Mixed infections (i.e. co-inoculation; with the panels of *gfp*, *mCherry* and *gfp-mCherry* merged images from left to right respectively); indicating the cell densities of QS ^+^ and QS^-^ cells of *Xcc* 8004/*gfp*, *Xcc* Δ*rpfF*/*mCherry* and *Xcc* Δ*rpfC*/*mCherry* bioreporter strains spanning transverse sections of leaf vascular regions on dpi 1, 6 and 12 (from top to bottom). Images were prepared using FIJI (image J) software. Scale bars on each panel, 5 μm. **(B)** Quantification of QS ^+^ and QS^-^ bacterial populations for single and mixed infections per cm^2^ proximal regions of inoculated leaves on dpi 1, 6 and 12. **(C)** Quantification of percentage survival of QS ^+^ and QS^-^ bacterial populations for mixed infection per cm^2^ proximal region of inoculated leaves on dpi 1, 6 and 12. WT; wild-type *Xcc* 8004, Δ*F*; *Xcc* Δ*rpfF*, and Δ*C*; *Xcc* Δ*rpfC*. The bacterial population size observed was normalized; values are expressed per cm^2^ leaf region. Bacterial fluorescence and quantification were analysed from CLSM images using FIJI (image J) software. The characteristics of the total region of the leaf observed on each sampling day were slightly different. Data analysis was done by taking six different sites from three infected leaves as samples for each combination at a time with the experimental repeat of at least thrice and represented with Mean ± SD. P-values for significant difference level were determined by performing student’s T-test (two tailed, paired). ***; p < 0.001.

Analysis of single and mixed infections (from CLSM studies) with the constitutive reporter strains of QS proficient wild-type *Xcc* 8004, its QS deficient Δ*rpfF* and Δ*rpfC* mutants indicated the reduced fitness in case of both the QS deficient Δ*rpfF* and Δ*rpfC* populations compared to QS proficient wild-type *Xcc* 8004 population during disease establishment *in planta*. However, in the mixed infection studies, both the QS null Δ*rpfF* and QS blind Δ*rpfC* mutants exhibited a significant reduction in fitness at later stage of disease establishment (12 dpi) (Fig 5A and 5B, S9A and S9B Fig). In the mixed infections, the wild-type QS responders outcompeted both the QS null Δ*rpfF* as well as QS blind Δ*rpfC* mutants towards *in planta* growth/localization separately, to exhibit a typically heterogeneous population with approximately 20% QS non-responders on 6 dpi and a more homogeneous population with only approximately 2% QS non-responders on 12 dpi for each combination (Fig 5C, S9C Fig). Furthermore, our *in planta* competition assay by CFU analysis for the above bacterial populations isolated from surface sterilized infected cabbage leaves on dpi(s) 1, 6 and 12 further corroborates with our *in planta* competition assays by CLSM analysis, indicating the significant QS-response benefits towards the survival fitness in case of QS proficient wild-type *Xcc* 8004 population as compared to its QS deficient Δ*rpfF* and Δ*rpfC* mutant populations on dpi(s) 6 and 12 (S10 Fig).

**Fig 6:**
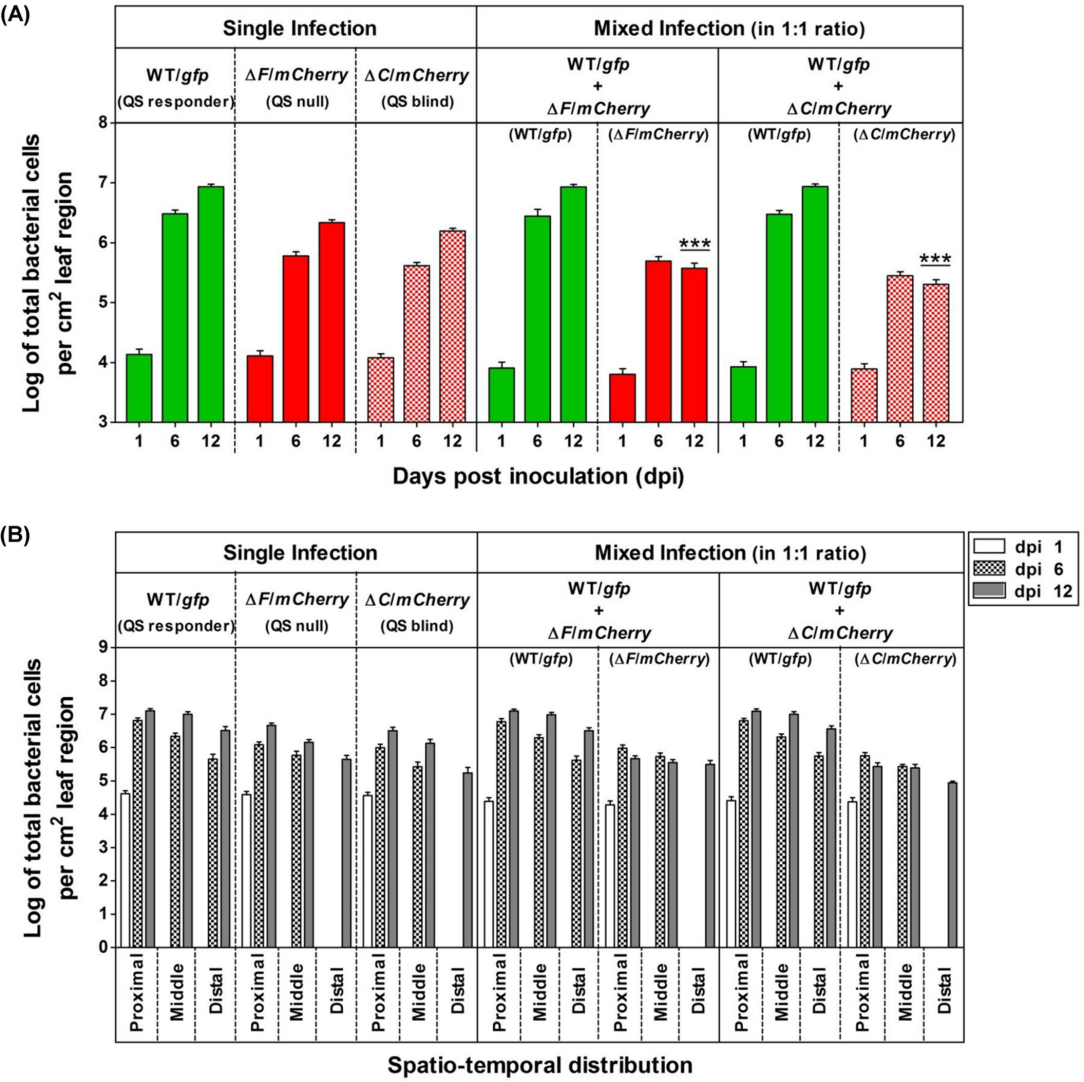
QS-response maximizes the *in planta* migration rate in *Xcc* for their better survibility. Frequency distribution of QS^+^ and QS^-^ bacterial populations in the *in planta* competition assay, indicating the growth and migration patterns for QS responder (i.e. *Xcc* 8004/*gfp*), QS null (i.e. *Xcc* Δ*rpfF*/*mCherry*) and QS blind (i.e. *Xcc* Δ*rpfC*/*mCherry*) cells for single as well as mixed infections within clip inoculated cabbage leaves under a CLSM upto dpi 12. **(A)** Average bacterial population size per 1 cm^2^ leaf regions, and **(B)** Spatio-temporal distribution of bacterial population size per 1 cm^2^ proximal, middle and distal leaf regions respectively, for single and mixed infections on dpi 1, 6 and 12. WT; wild-type *Xcc* 8004, Δ*F*; *Xcc* Δ*rpfF*, and Δ*C*; *Xcc* Δ*rpfC*. On specified sampling dpi, multiple Z-stalks were acquired under a CLSM for each sample under green and red fluorescence along with DIC channel, maintaining 0.5 μm gap between two successive Z-planes. Bacterial population size was analysed by considering the X,Y and Z planes for each Z-stalk, where the bacterial cells present in all the Z-planes were counted manually and summed up to calculate the total no. of bacterial cells in that region at a time. The total population size observed was normalized; values are expressed per cm^2^ leaf region. The bacterial population size for each infection was determined by combining the analysed data for five sites per inoculated leaf, six leaves on each sampling day with experimental repeats for thrice. The characteristics of the total region of the leaf observed on each sampling day were slightly different. Data analysis [using FIJI (image J) software] was performed by taking six different confocal images as samples for each strain at a time with the experimental repeat of at least thrice and represented with Mean ± SD. P-values for significant difference level were determined by performing student’s T-test (two tailed, paired). ***; p < 0.001.

The analysis of frequency distribution of QS^+^ and QS^-^ bacterial populations in the *in planta* competition assay indicated sufficiently higher population size in case of QS^+^ wild-type *Xcc* 8004 (i.e. ~ 5 to 10 fold higher) compared its QS null Δ*rpfF* and QS blind Δ*rpfC* mutants on dpi(s) 6 and 12. The average population size of wild-type *Xcc* 8004 bioreporter cells reached quickly upto ~ 3 × 10^6^ cells per cm^2^ leaf region to develop the disease symptoms within the inoculated leaves on dpi 6, and also attended maximum population size of ~ 10^7^ cells per cm^2^ leaf region within infected cabbage leaves as compared to the populations of its QS null Δ*rpfF* (i.e. ~ 2 × 10^6^ cells per cm^2^ leaf region) and QS blind Δ*rpfC* mutants (i.e. ~ 1.5 × 10^6^ cells per cm^2^ leaf region) on dpi 12. However, the population sizes for QS null Δ*rpfF* and QS blind Δ*rpfC* mutants were found to be significantly reduced in the presence of QS^+^ wild-type *Xcc* 8004 cells at the late stages of disease establishment (i.e. from dpi 6 to 12) in the co-infected cabbage leaves (Fig 6A, S11A Fig). Further, analysis of frequency distribution of QS^+^ and QS^-^ bacterial populations was carried out to understand the QS benefits towards *in planta* migration and spatio-temporal regulation of population size in *Xcc*. On specified sampling dpi(s), analysis of population distribution patterns within proximal, middle and distal regions of inoculated leaves with single cultures revealed significantly higher population size per cm^2^ infected leaf region in case of wild-type *Xcc* compared to the QS null Δ*rpfF* and QS blind Δ*rpfC* mutant populations from dpi(s) 6 (~ 5–7 folds higher than Δ*rpfF*, ~ 7–11 folds higher than Δ*rpfC*) to 12 (~ 4–5 folds higher than Δ*rpfF*, ~ 5–7 folds higher than Δ*rpfC*). However in the mixed infections, there was a drastic reduction in the population size for both the QS null and QS blind mutants in the presence of QS-responding wild-type *Xcc* (~ 23–33 folds lower for Δ*rpfF*, ~ 43–62 folds lower for Δ*rpfC*) on 12 dpi. Detailed analysis indicated a drastic reduction in bacterial population size in case of QS blind Δ*rpfC* mutant population compared to both QS-responsive wild-type *Xcc* 8004 as well as QS null *Xcc* Δ*rpfF* mutant populations spanning all the proximal, middle and distal regions upto dpi 12, and the bacterial population size was found to be maximum and minimum within proximal and distal regions respectively (Fig 6B, S11B Fig).

**Fig 7:**
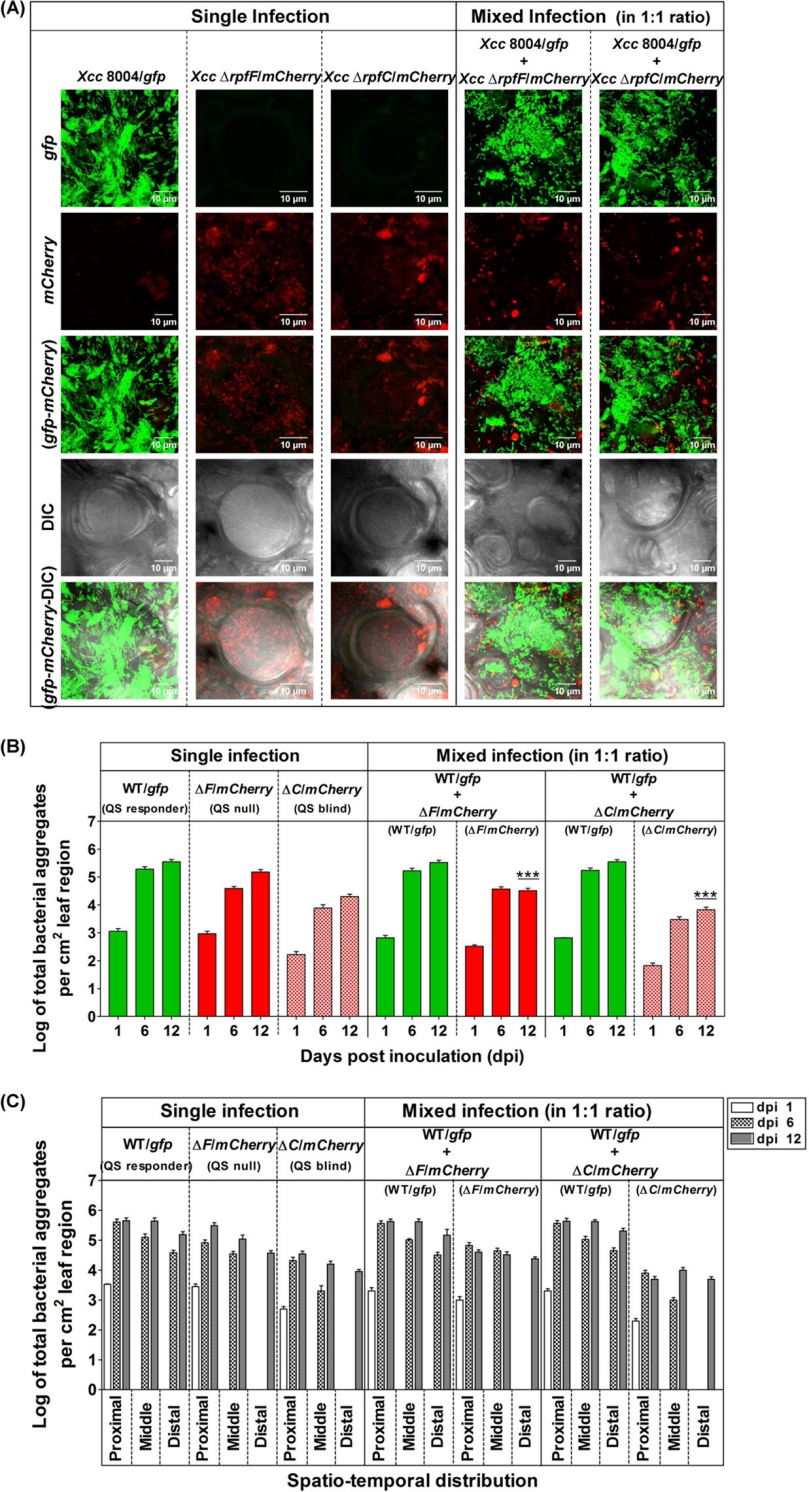
Reduced cell-aggregation in QS mutants cannot be rescued by QS responders of *Xcc in planta*. **(A)** Representative dpi 12 CLSM images for cell aggregate formation patterns of QS responder (i.e. *Xcc* 8004/*gfp*), QS null (i.e. *Xcc* Δ*rpfF*/*mCherry*) and QS blind (i.e. *Xcc* Δ*rpfC*/*mCherry*) cells for single as well as mixed infections as specified combinations (panels from left to right) in the *in planta* competition assay within proximal vascular regions of clip inoculated cabbage leaves under a CLSM upto dpi 12. The panels from top to bottom show *gfp*, *mCherry*, *gfp*-*mCherry* merged, DIC and *gfp*-*mCherry-*DIC merged images for both single and mixed infections respectively. Images were prepared using FIJI (image J) software. Scale bars on each panel, 10 μm. **(B)** Average no. of bacterial aggregates per 1 cm^2^ of total leaf regions, and **(C)** Spatio-temporal distribution of no. of bacterial aggregates per 1 cm^2^ proximal, middle and distal leaf regions respectively, for single and mixed infections on dpi 1, 6 and 12. WT; wild-type *Xcc* 8004, Δ*F*; *Xcc* Δ*rpfF*, and Δ*C*; *Xcc* Δ*rpfC*. On specified sampling dpi, multiple Z-stalks were acquired under a CLSM for each sample under green and red fluorescence along with DIC channel, maintaining 0.5 μm gap between two successive Z-planes. Bacterial aggregate size as well as no. were analysed by considering the X,Y and Z planes for each aggregate of a Z-stalk, where the bacterial aggregates present in all the Z-planes were counted manually and summed up to calculate their total no. in that region at a time. Total bacterial aggregate no. observed was normalized and the values are expressed per cm^2^ leaf region. The no. of bacterial aggregates for each infection was determined by combining the analysed data for five sites per inoculated leaf, six leaves on each sampling day with experimental repeats for thrice. The characteristics of the total region of the leaf observed at each sampling time were slightly different. Data analysis [using FIJI (image J) software] was performed by taking six different confocal images as samples for each strain at a time with the experimental repeat of at least thrice and represented with Mean ± SD. P-values for significant difference level were determined by performing student’s T-test (two tailed, paired). ***; p < 0.001.

To understand the QS-regulated cell aggregate formation *in planta* during disease establishment, we have also analyzed the frequency distribution of aggregate numbers as a function of time in the single and mixed infections. Confocal microscopy of infected cabbage leaves at late stage of disease establishment indicated the presence of significantly higher no. of larger bacterial aggregates in the bioreporter populations of wild-type *Xcc* 8004 as compared to its QS null Δ*rpfF* and QS blind Δ*rpfC* mutants within proximal vascular regions on dpi 12 (Fig 7A). On specified sampling dpi(s), analysis of aggregate formation patterns within proximal, middle and distal regions of inoculated leaves with single cultures revealed significantly higher no. of aggregate formation per cm^2^ infected leaf region in case of wild-type *Xcc* compared to the QS null Δ*rpfF* and QS blind Δ*rpfC* mutant populations from dpi(s) 6 (~ 5 folds higher than Δ*rpfF*, ~ 24–38 folds higher than Δ*rpfC*) to 12 (~ 2.5 folds higher than Δ*rpfF*, ~ 18–21 folds higher than Δ*rpfC*). However in the mixed infections, there was a drastic reduction in the number of bacterial aggregates for both the QS null and QS blind mutants in the presence of QS-responding wild-type *Xcc* (~ 10–14 folds lower for Δ*rpfF*, ~ 53–67 folds lower for Δ*rpfC*) on 12 dpi (Fig 7B, S12A Fig). Detailed analysis indicated a drastic reduction in the no. of bacterial aggregates in case of QS blind Δ*rpfC* mutant population compared to both QS-responsive wild-type *Xcc* 8004 as well as QS null *Xcc* Δ*rpfF* mutant populations spanning all the proximal, middle and distal regions upto dpi 12, and the frequency towards larger aggregate formation for each population was found to be maximum and minimum within proximal and distal regions respectively (Fig 7C, S12B Fig).

**Fig 8:**
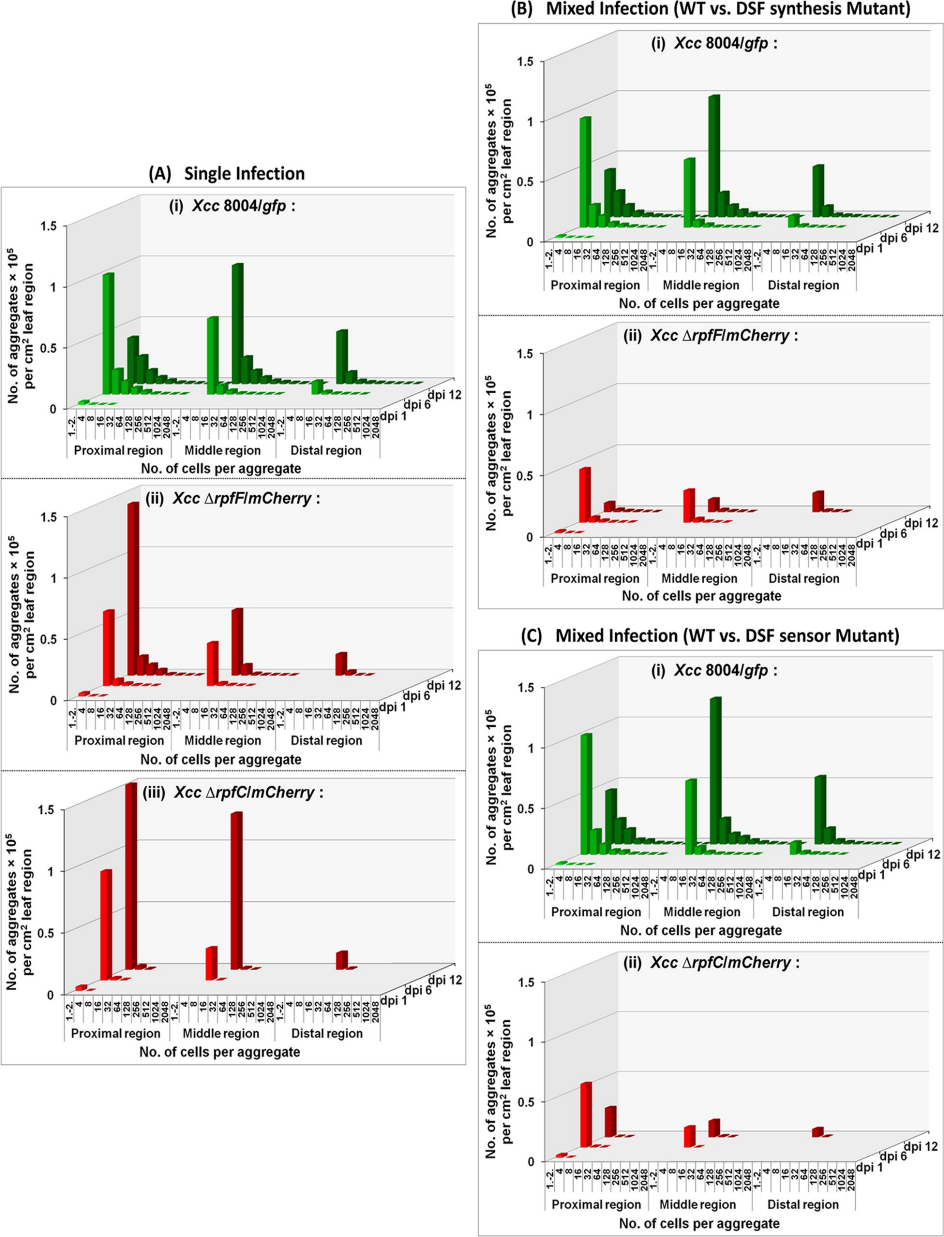
Frequency distribution of bacterial aggregates of *Xcc* populations *in planta*. **(A)** Single Infection studies; with (i) *Xcc 8004*/*gfp* (WT), (ii) *Xcc* Δ*rpfF*/*mCherry* (DSF synthesis mutant) and (iii) *Xcc* Δ*rpfC*/*mCherry* (DSF sensor mutant), **(B)** Mixed Infection studies; with (i) *Xcc 8004*/*gfp* (WT) vs. (ii) *Xcc* Δ*rpfF*/*mCherry* (DSF synthesis mutant), and **(C)** Mixed Infection studies; with (i) *Xcc 8004*/*gfp* (WT) vs. (ii) *Xcc* Δ*rpfC*/*mCherry* (DSF sensor mutant). On specific dpi, the bacterial aggregates were observed and their frequency distribution were calculated within proximal, middle and distal green regions to the infection site spanning approximately 1cm distance for each region. The total number of bacterial aggregates for each infection was determined for six leaves at each sampling time, and the data were combined. The characteristics of the total region of the leaf observed at each sampling time were slightly different. The number of cells per aggregate was estimated from the surface area and depth of each aggregate using FIJI (image J) software. The total number of aggregates observed was normalized; values are expressed per cm^2^ leaf region.

Furthermore, we analyzed the frequency of distribution of bacterial aggregate size as a function of time in the single and mixed infection studies. Larger aggregates were observed for the wild-type *Xcc* population rather than the QS null and QS blind mutants from 6 dpi onwards. Between 6 to 12 dpi, significantly higher number of large size bacterial aggregates was observed for wild-type *Xcc* population within the proximal regions, however, on 12 dpi, more number of solitary and comparatively smaller size bacterial aggregates was observed within the middle regions as compared to proximal regions. Both, QS null Δ*rpfF* as well as QS blind Δ*rpfC* mutant populations exhibited a significant reduction in the number as well as in size of bacterial aggregates and migration in the presence of QS proficient wild-type *Xcc* 8004 population on 12 dpi within the proximal, middle and distal region from the point of inoculation in the cabbage leaves (Fig 8, S13 Fig).

## Discussion

Quorum sensing plays an important role in the virulence of several plant and animal pathogenic bacteria by coordinating the production of different sets of virulence associated factors via synchronizing gene expression, in a density dependent fashion [1, 2, 3, 4]. However, an increasing body of research suggests that bacteria exhibit non-genetic phenotypic heterogeneity in the QS-response within the isogenic bacterial population under homogeneous laboratory culture conditions [8, 9, 10, 11]. However, little is known about the nature of phenotypic heterogeneity in the QS-response and its role in cooperative behavior in natural environment such as inside the host.

Pathogenic bacteria depend quite significantly on QS regulation to coordinate their colonization and infection of plant hosts [2]. DSF family mediated QS-response regulates diverse virulence factors towards *Xanthomonas* virulence in natural host plant [15, 16]. Within host plant, initially the pathogen at a low cell density escapes the host immune system by not performing QS. However, upon achieving a certain cell density the bacterial population activates the QS circuit to maximize its *in planta* fitness via exhibiting stochastic phenotypic heterogeneity within the host plant [14, 15, 19].

Recent *in vitro* studies of QS-response at the single cell level in *Xanthomonas campestris* pv. *campestris* and *Pseudomonas syringae* have indicated that bacteria maintain QS-responsive and non-responsive sub-populations in a ~ 80:20 ratio independent of their origin, even at high cell density and in the presence of excess of exogenously supplemented QS signal. The mixed motility assay indicated that the presence of both responding and non-responding cells could serve as a bet hedging strategy, thus promoting QS-responsive cells for more spread inside the vessel and non-responsive cells to utilize local host resources [9]. However, it was unclear whether the inherent stochastic heterogeneity in the QS-response exhibited under laboratory condition is influenced by change in environmental conditions, and whether there is selection pressure to cooperate under natural conditions particularly in host-pathogen interaction. In the present study, we have now added a detail statement about the lifestyle of the pathogen in which QS-regulated virulence associated functions are involved in adaptation of different stages of infection in its host plant.

Our *in planta* results indicated that the plant pathogen *Xcc* exhibits heterogeneity in the QS-response with bimodal QS distribution in its population at early stage of disease establishment, with the occurrence of both responding and non-responding cells. In contrast to earlier studies, the studies presented here argue that heterogeneity in QS-response is not due to the lack of cross-induction which may arise due to spatial structures that could limit sharing of public goods such as the QS signal among the members of the community, as QS-responsive and non-responsive cells coexist together in similar size aggregates or microcolonies inside the host plant [10, 19]. However, during the later stages of the infection, the wild-type *Xcc* exhibited a synchronized homogeneous QS-response with almost all viable cells to be QS-induced state. In this study, we have shown that the QS-response benefits towards *in planta* survival fitness of QS responders over QS non-responders as a potential regulator to interplay between heterogeneity and homogeneity towards QS-response within *Xcc* population at sufficiently high cell density under nutrient scarce conditions inside the host plants. Based on our recent results, here we argue that this interplay between heterogeneity and homogeneity towards QS-response inside the host plants could provide a stage specific adaptive advantage to the bacterial populations towards successful utilization of environmental resources, which in turn helps them to adapt to changing environmental condition.

Our current QS induction response dynamics studies indicated towards the existence of bimodal QS distribution with the heterogeneously QS-induced *Xcc* populations *in vitro* (S2 and S5 Figs) as well as *in planta* (Figs 1, 2 and 3). The quorum size within *Xcc* aggregate was highly influenced by the aggregate size spatio-temporally within host plant leaves. The existence of QS heterogeneity even within larger bacterial aggregates (~ 10^3^ cells per aggregate) during 3^rd^ to 10^th^ dpi indicates that, the QS non-responders are unable to share the QS benefits in presence of QS responders without any spatial restriction for QS distribution *in planta* (Fig 4).

In our dual-bioreporter based *in planta* studies (Figs 2, 3 and 4), the absence of QS non-responders in wild-type *Xcc* dual-bioreporter population at the late stage of infection (on dpi 12) indicated towards the non-sharing of QS benefits towards social co-operation under unfavorable conditions, such as nutrient limitation in natural host plant. The inability of QS non-responders of *Xcc* to exhibit the QS-response even in presence of excess signal under artificial laboratory conditions [9] discards the possibility of QS-response by those non-responders in the homogeneously QS-induced population at late stage of infection at high concentration of quorum signal *in planta*. Hence, we hypothesized that the non-sharing of QS benefit could be driving force towards the selective fitness of QS responders over QS non-responders under unfavorable plant host environment. To further prove this hypothesis, an *in planta* competition assay was performed using single constitutive reporter strains (Fig 5), where we have used the QS-responsive wild-type *Xcc* along with its Δ*rpfF* (DSF null) and Δ*rpfC* (DSF blind) mutants as QS negative controls. It is known that QS mutants (Δ*rpfF* and Δ*rpfC*) in *Xcc* are growth deficient with compromised fitness as compared to the QS performing wild-type cells *in planta* [28]. Through our *in planta* competition assay, we wanted to find out whether the QS benefits can be shared by QS mutants in the presence of QS performers (without any spatial restriction towards QS distribution as mentioned in earlier studies [11, 19] to rescue their *in planta* fitness.

In earlier studies, the QS mutants of *Xylella fastidiosa* and *Ralstonia solanacearum* exhibit significant fitness defects in associating with their insect and plant hosts respectively [29, 30]. However, our recent *in planta* competition assays with the QS-responding wild-type, its QS null (DSF synthase) and QS blind (DSF sensor) mutant strains indicated that although QS mutants and wild-type cells co-exist together sharing common micro-niche inside the host plant, both the QS null and blind mutants exhibited significant retardation in growth (Fig 5), migration and survival (Fig 6) and cell-aggregate formation (Figs 7 and 8) in the presence of wild-type, particularly at the late stage of the disease. This suggests that the declined in fitness of QS non-responders in the presence of QS responders may be the reason for a homogeneously QS-induced population at high cell density during late stages of disease establishment) in host plant. These results contrast with earlier report where it has been shown that the QS cheats or non-responders have fitness cost in the presence of wild-type due to spatial constrain, as QS non-responders and wild-type cells form well separated and discrete microcolonies inside the host which results in non sharing of public goods [19].

**Fig 9:**
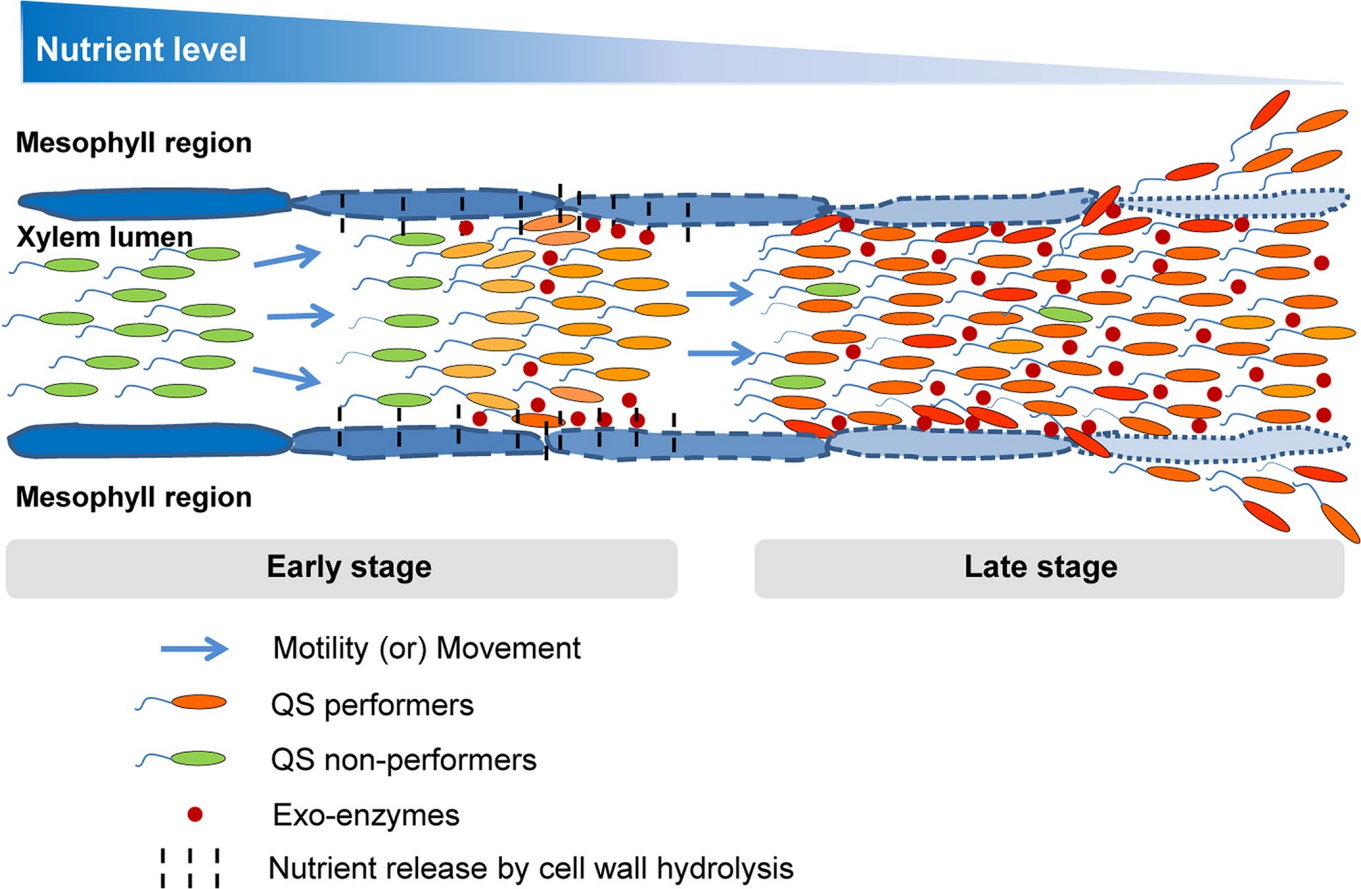
A proposed model for the interplay of heterogeneity and homogeneity in bacterial QS-response in infection. In *Xanthomonas campestris* pv. *campestris* QS positively regulate motility, extracellular enzyme (public good) production and promote bacterial dispersal *in planta*. At the early stage of disease, along with bacterial growth and dispersal within the infected xylem lumens, heterogeneous QS induction happens locally in the sufficiently grown bacterial population, where the QS responders produce exo-enzymes to degrade the surrounding plant cell wall to escape the bacteria into surrounding mesophyll regions and promote systemic spread. In contrast, the QS non-responders utilize nutrients released by the action of exo-enzymes produced by responders. As bacterial density increase at the late stage of disease the QS responders out compete QS non-responder cells as there is nutritional limitation, which may cause growth disadvantage for non-responders which are deficient in spreading to new nutrient rich niche.

Here, we propose that the pathogen interplays between non-genetic heterogeneity and homogeneity towards QS-response spatio-temporally for their better survival and successful disease establishment in host plant. The idea is that, at the early stage of disease, presumably under nutrient sufficient condition, QS-responsive cells contribute to spread and establishment of systemic infection. The QS non-responsive cells contribute more towards colonization and utilization of resources locally. However during the later stage of disease, presumably under condition of nutrient limitation due to the large increase in bacterial load, bet-hedging may be disadvantageous as the free-loaders share the limited resources. At this stage, QS-responsive cells have growth advantage probably by the production of ‘private goods’ [18] required for survival under these condition (Fig 9). In other words, we assume that during *in planta* proliferation, the part of the over-saturated bacterial population lagging behind in migration experiences a severe nutrient scarcity (referred as “nutrient limitation”) locally. Under such nutrient limitation, iron [31], nitrogen and phosphorus sources also get depleted along with total carbon (as a major nutrient) locally [32], and there is a decrease in the availability of such resources for QS non-responders as compared to QS responders within this part of the obove population. As a result, the QS non-responders experience comparatively higher nutrient limitation over a time period and gradually get eliminated out from the population locally.

Previously, we have shown that under *in vitro* laboratory conditions, *Xcc* exhibit stochastic heterogeneity in QS-response with the distribution of both QS-responsive and non responsive cell approximately in an 80:20 ratio at high cell density. Exogenous addition of excess QS signal DSF did not alter the distribution of QS-responsive and non-responsive sub-populations. The fact that an *E*. *coli* strain harbouring the QS-responsive signalling components exhibited unison response in the presence of exogenous QS signal molecule, indicating that the QS-responding bacteria in general exhibit inherent stochastic phenotypic heterogeneity in QS-response [9]. It is likely that under natural conditions; such as later stages of disease progression *in planta*, there is selective advantage of QS-responsive sub-population as evident in our *in planta* competition assays, co-inoculated with wild-type *Xcc* 8004 and either its DSF null (or) DSF blind mutants separately.

Interestingly, it has been shown that *Salmonella typhimurium*, a human pathogen exhibited phenotypic heterogeneity in production of virulence factors which are required for host colonization, are expressed in a bistable fashion, leading to sub-populations of virulent and avirulent cells in the population [33]. It has been proposed that the heterogeneity in production of virulence factor functions gives stability to the population as whole, as the non-producers have growth advantage that could limit spontaneous occurrence of cheaters in the population, which could be more deleterious [33]. It is pertinent to note that in *Xanthomonas*, it has been reported that during stationary phase, extracellular polysaccharide deficient mutants arise spontaneously in the wild-type population due to transposition of the endogenous transposon in the EPS biosynthetic genes [34]. In *Xanthomonas*, DSF is also involved in the regulation of production of EPS. Therefore, it is possible that maintaining phenotypic heterogeneity in QS-responding population could possibly also limit the spontaneous occurrence of EPS deficient mutants *in planta* which could possibly affect virulence by the sharing or utilization of recourse produced by the QS-responding population.

Taken together, our results indicate that interplays between QS heterogeneity and homogeneity at specific stages of infection maximize the phytopathogenic bacterial population fitness under changing environmental conditions in host plant and hence to cause successful disease establishment.

## Materials and methods

### Bacterial strains and growth conditions

*Xcc* 8004 and its derived strains were maintained on Peptone Sucrose Agar (PSA) and grown in PS broth at 28°C with 200 rpm, as described previously [35, 36]. For *in vitro* QS induction experiment, the exponential phase 1^o^ cultures were sub-cultured into fresh PS broth and grown upto 44 hrs. For all plant infection experiments, the exponential phase 1^o^ cultures were sub-cultured and grown upto 10^7^ cells per ml at 28°C. The *Escherichia coli* DH5α and its derived strains used for routine cloning were maintained on Luria-Bertani Agar (LBA) and grown in LB broth [35] at 37°C with 200 rpm. The concentrations of the appropriate antibiotics used were rifampicin (Rif; 50 μg/ml), ampicillin (Amp; 400 μg/ml or 100 μg/ml), nalidixic acid (Nal; 50 μg/ml) and 5-bromo-4-chloro-3-indolyl-D-galactoside (X-Gal; 25 μg/ml).

### Generation of DSF responsive reporter strains of *Xcc*

Standard molecular biology and microbiology techniques were employed for generating different *Xcc* derived reporter strains, as mentioned earlier [31]; where, different transcriptional fusions were constructed by fusing the promoter regions (*P*_*kan*_, *P*_*eng*_ or *P*_*lac*_) upstream of a gene of interest, to a fluorescent protein gene (*gfp*, *rfp*, *mCherry* or *gusA*), and cloned into either pBBR4 (*P*_*kan*_:*gfp*, *P*_*eng*_:*rfp*, *P*_*lac*_:*mCherry*) or pLAFR6 (*P*_*eng*_:*gusA*) plasmids.

The DSF responsive dual-reporter strains of *Xcc* {i.e. *Xcc* 8004 (pBBR4/*P*_*kan*_:*gfp*-*P*_*eng*_:*rfp*) and *Xcc* Δ*rpfF* (pBBR4/*P*_*kan*_:*gfp*-*P*_*eng*_:*rfp*)} were generated, where pBBR4 [37] harboured both a constitutive *gfp* marker gene and a DSF regulated *rfp* reporter gene. Briefly, the Kanamycin promoter (i.e. *P*_*kan*_) sequence (137 bp fragment) was amplified from EZ-Tn5 <KAN-2> Insertion Kit (Cat. No. EZI982K) with the forward primer (with EcoRI); 5’-GCGAATTCCTGTCTCTTATACACATC-3’ and reverse primer (with SalI); 5’-GCGTCGACAACACCCCTTGTATTAC-3’. The *gfp* coding sequence ORF (716 bp fragment) was amplified from pPROBE-GT plasmid with the forward primer (with SalI and universal *rbs* sequence before the START codon); 5’-GCGTCGAC*AGGAGGACAGCT*ATGAGTAAAGGAGAAGAA-3’ and reverse primer (with BamHI and STOP codon); 5’-GCGGATCCTCATTTGTATAGTTCATCCATG-3’. Ligation of 3’ end of *P*_*kan*_ with 5’ end of *gfp* ORF with the SalI restriction site followed by double digestion at the two ends of the ligated product formed the first constitutive *gfp* reporter cassette (a 853 bp EcoRI–BamHI fragment), which then was cloned into pBBR4 plasmid creating pBBR4/P_*kan*_:*gfp*. For the second DSF responsive *rfp* reporter cassette, the predicted Endoglucanase (XC_0639) promoter (i.e. *P*_*eng*_) sequence (372 bp fragment) was amplified from *Xcc* 8004 genomic DNA with the forward primer (with XhoI); 5’-GCCTCGAGTCACAAACGACGCGAACA-3’ and reverse primer (with EcoRI); 5’-GCGAATTCCATGGTGATCTCCCTAG-3’. The *rfp* coding sequence ORF (675 bp fragment) was amplified from pDsRed-monomer vector (Cat. No. 632467) with the forward primer (with EcoRI); 5’-GCGAATTCGACAACACCGAGGACGTCATC-3’ and reverse primer (with KpnI and STOP codon); 5’-GCGGTACCCTACTGGGAGCCGGAGTG-3’. Ligation of 3’ end of *P*_*eng*_ with 5’ end of *rfp* ORF with the EcoRI restriction site followed by double digestion at the two ends formed the second DSF responsive *rfp* reporter cassette (a 1053 bp XhoI–KpnI fragment) which then was cloned into pBBR4/*P*_*kan*_:*gfp* plasmid construct creating a dual construct (pBBR4/*P*_*kan*_:*gfp*-*P*_*eng*_:*rfp*), wherein the *P*_*kan*_:*gfp* and *P*_*eng*_:*rfp* cassettes were divergent. After further confirming each cassette’s orientation by sequence analysis, the dual reporter construct (pBBR4/*P*_*kan*_:*gfp*-*P*_*eng*_:*rfp*) was then introduced into *Xcc* 8004 and its DSF deficient Δ*rpfF* strain by electroporation resulting *Xcc* 8004 (pBBR4/*P*_*kan*_:*gfp*-*P*_*eng*_:*rfp*) and *Xcc* Δ*rpfF* (pBBR4/*P*_*kan*_:*gfp-P*_*eng*_:*rfp*) separately. Initial screening for *Xcc* 8004 (pBBR4/*P*_*kan*_:*gfp*-*P*_*eng*_:*rfp*) and *Xcc* Δ*rpfF* (pBBR4/*P*_*kan*_:*gfp-P*_*eng*_:*rfp*) strains were performed by visualizing their optimally grown cultures in nutrient rich PS media (supplemented with required antibiotics) for their GFP and RFP fluorescence using confocal laser-scanning microscopy (CLSM); where GFP was excited at 488 nm and the fluorescence was collected in the range of 505–550 nm (filter set 38 HE eGFP, Zeiss), and RFP was excited at 555 nm and the fluorescence was collected in the range of 582–800 nm (filter set 20 Rhodamin, Zeiss).

In addition, the DSF responsive GUS reporter strain *Xcc* 8004 (pLAFR6/*P*_*eng*_:*gusA*) were also constructed, harbouring DSF responsive *gusA* marker gene within a stable plasmid pLAFR6 [38]. Construction of the DSF responsive GFP reporter strain *Xcc* 8004 (pKLN55/*P*_*eng*_:*gfp*) used in this study has been previously described [6]. Other reporter strains *Xcc* 8004 (pBBR4/*P*_*kan*_:*gfp*), *Xcc* Δ*rpfF* (pBBR4/*P*_*kan*_:*gfp*), *Xcc* Δ*rpfC* (pBBR4/*P*_*kan*_:*gfp*), *Xcc* 8004 (pBBR4/*P*_*lac*_:*mCherry*), *Xcc* Δ*rpfF* (pBBR4/*P*_*lac*_:*mCherry*) and *Xcc* Δ*rpfC* (pBBR4/*P*_*lac*_:*mCherry*) were generated; harbouring either constitutive *gfp* or *mCherry* marker genes in stable plasmid pBBR4. All the plasmids are low copy number, and were stably maintained during infection. Confocal microscopy as well as GUS assay was performed to screen and demonstrate the reporter expression the newly generated reporter strains in broth cultures.

All the newly constructed *Xcc* bioreporter strains were checked for *in vitro* and *in planta* growth. The *in planta* and/or *in vitro* growth assay with different bioreporter constructs revelled similar growth pattern with either the GFP, RFP and m-Cherry based reporters as compared to the respective strains of *Xcc* without those reporter constructs.

### *In vitro* GUS assay (for initial screening of *gus* reporter strains of *Xcc*)

*In vitro* GUS assay was performed to screen the GUS reporter strains of *Xcc*. Briefly, the wild-type GUS reporter strain *Xcc* 8004 (pLAFR6/*P*_*eng*_:*gusA*) was grown along with *Xcc* Δ*rpfF* (pLAFR6/*P*_*eng*_:*gusA*) as its QS negative control strain separately in nutrient rich PS media with the appropriate antibiotics at 28°C and 200 rpm overnight. After appropriate OD normalization, 0.2% of primary inoculum for each culture was transferred into nutrient rich fresh PS media and incubated at 28°C and 200 rpm upto 44 hrs. The absorbance at 600 nm and GUS expression were measured at regular time intervals of 12 hrs. GUS expression assays were performed as described previously [39] with a few modifications. Briefly, cells were harvested from 1 ml of culture aliquot by centrifugation at 5000 rpm for 6 min (New Brunswick Scientific, Innova 43, Edison, NJ, USA) for specified time period, followed 0.2% NaCl wash of the cell pellet for twice. Pellets were resuspended in 250 μl extraction buffer [50 mM sodium di-hydrogen phosphate (pH 7.0), 10 mM ethylene di-amine tetra acetic acid (EDTA), 10 mM β-mercaptoethanol, 0.1% Triton X-100 and 0.1% sodium lauryl sarcosine] with added 1 mM MUG (4-methylumbelliferyl β-D-glucuronide) and incubated at 37°C. After a definite time interval (i.e. 30 mins of incubation), reactions were terminated by the addition of 675 μl of 0.2 M Na_2_CO_3_ into 75 μl of reaction mixture. Fluorescence was measured with 4-methylumbelliferone (4-MU; Sigma) as standard at an excitation wavelength of 365 nm and emission wavelength of 455 nm. GUS activity was presented as nano moles of 4-MU produced per minute.

### CLSM study and CFU assay to visualise QS Induction in *Xcc* at different cell densities *in vitro*

The *Xcc* 8004 dual-bioreporter strains *Xcc* 8004 (pBBR4/*P*_*kan*_:*gfp*-*P*_*eng*_:*rfp*) and *Xcc* Δ*rpfF* (pBBR4/*P*_*kan*_:*gfp-P*_*eng*_:*rfp*) were grown in the liquid PS broth with the respective antibiotics upto a cell concentration (i.e. 10^7^ cells per ml) at which QS induction has yet to occur. 0.2% (v/v) inoculum of this primary culture was used for the 2^o^ cultures to attain an initial culture density of ~ 6 × 10^4^ cells ml^-1^, followed by its incubation at 28°C with 200 rpm upto 44 hrs. For the DSF supplementation, the extracted *Xoo* DSF as well as commercial DSF (dissolved in ethyl acetate) were placed in glass culture tubes, air-dried, and resuspended with fresh PS broth to final concentration of 4.84μM (i.e. optimal concentration; that is the threshold amount of DSF required to phenocopy the wild-type *Xcc* towards QS induction in its Δ*rpfF* culture) separately, followed by addition of the 2^o^ inocula of the *Xcc* Δ*rpfF* (pBBR4/*P*_*kan*_:*gfp-P*_*eng*_:*rfp*) strain. The bacterial cells from 1ml of 2^o^ culture aliquotes were harvested in triplicates at specific time intervals upto 44 hrs, by centrifugation at 5000 rpm for 6 min (New Brunswick Scientific, Innova 43, Edison, NJ, USA) followed 0.2% NaCl wash of the cell pellet for twice and re-constitution in sterile PBS (1X, pH 7.4) buffer. Approximately, 8 μl of each sample was mounted on glass slide (Rohem Industries pvt. Ltd; IS-3099) at each at each sampling time and observed using a confocal laser-scanning microscope under 100x/1.4 oil DIC M27 objective (LSM700; Carl Zeiss, Germany) for the expression of both *gfp* (excitation: 488 nm and emissions: 505 to 550 nm band pass, with 518 nm emission maximum) and *rfp* (excitation: 555 nm and emissions: 582 to 800 nm band pass, with 585 nm emission maximum) reporter genes in wild-type *Xcc* 8004 along with its DSF synthesis mutant, *Xcc* Δ*rpfF* (with and without initial supplementation with external DSF to the culture). Multiple images were acquired using green and red fluorescence and bright field (DIC) for each slide. The actual QS-induced RFP fluorescence intensities for both the wild-type *Xcc* 8004 as well as its Δ*rpfF* (supplemented with 4.84μM external DSF) were calculated by subtracting the background RFP fluorescence intensities of *Xcc* Δ*rpfF* for basal level promoter expression at mentioned time periods with 4 hrs intervals upto 44 hr of growth. Confocal images for GFP (green), RFP (red) and Differential Interference Contrast (DIC) were constructed simultaneously using a multitrack mode via Pigtail-coupled solid-state lasers. Outlines of the individual bacterial cells were recognized form the DIC images for each time point. Approximately 400 to 600 cells per sample were analyzed for both GFP and RFP fluorescence patterns (approximately 70 to 100 cells per field were observed for 5 different fields) with experimental repeats for at least thrice.

Simultaneously, appropriate concentrations of 100 μl sample from the 1 ml culture aliquotes for each strain was dilution plated on the nutrient rich solid PSA medium supplemented with suitable antibiotics to determine the bacterial cell density in terms of CFUs per ml for each culture at each sampling time.

At each time point, the samples were observed under CLSM for the constitutive *gfp* and DSF responsive QS-regulated *rfp* expression by acquiring multiple images using green and red fluorescence for each strain at different cell densities (CFU/ml) throughout their growth *in vitro*.

### Cabbage leaf clip inoculation with *Xcc* bioreporter cells

The 2^o^ cultures of *Xcc* bioreporter strains were grown to a cell density of 10^6^ cells ml^-1^ and the bacterial cells in 1 ml of culture aliquot were harvested by centrifugation at 5000 rpm for 6 min (New Brunswick Scientific, Innova 43, Edison, NJ, USA), and reconstituted in sterile PBS (1X, pH 7.4) buffer. The appropriate bacterial suspensions (approximately 20 μl per leaf) of QS uninduced cells were then clip inoculated with the help of sterilized scissors into 40 days old healthy cabbage (*Brassica oleracea*) cultivar (Super Ball; Indian F1 Hybrid variety) by gently incising at the apex area of the healthy leaves (5–6 leafs per plant, total 6 plants). Cabbage plant inoculated with sterile PBS (1X, pH 7.4) buffer was used as a negative control. In order to facilitate the initial survival and growth of *Xcc* on leaves, the inoculated cabbage plants were placed in plant growth chamber (Adaptis by Conviron; CMP 6010) at 28°C with ambient R.H. (65% R.H.), where artificial light was maintained for 10 hr periods within the 24 hr post inoculation, and then removed from the chamber and kept under natural condition throughout the experiment.

### *In planta* competition assays

Briefly, 40 days old healthy cabbage plants were infected by clip inoculating the leaves with co-cultures (in 1:1 ratios, from ~ 10^6^ cells ml^-1^ 2^o^ culture) of wild-type *Xcc* 8004 (expressing constitutive *gfp*) in combinations with, either wild-type *Xcc* 8004 (or) its Δ*rpfF* (or) its Δ*rpfC* (each one expressing constitutive *mCherry*) along with their single cultures separately. In parallel, the obove experimental repeats were also performed with the reciprocal reporter constructs for each reporter strain to rule out the possible differential survival fitness effects due to different reporter constructs. On specified sampling dpi upto 12 days post inoculation, the proximal green regions spanning the mid-rib (upto immediate 1 cm distance from the clipped site excluding diseased part) were observed under a CLSM with 100x objective along with *in planta* CFU assay to analyse the bacterial colonization within the specific host tissue.

### Confocal Laser-Scanning Microscopy (CLSM) for *Xcc* bioreporter cells within leaves

Inoculated leaves from different cabbage plants (at least five leaves per plant) were examined upto 12 days after inoculation to visualize both localization and QS induction (in case of DSF responsive single and dual-bioreporter strains of *Xcc*, along with their negative control strains), as well as both localization and migration (in case of constitutive single bioreporter strains of *Xcc*). On each sampling dpi, the leaves were collected immediately prior to sectioning and sample preparation and the transverse sections of leaf slices were observed under a CLSM with 100x objective for its vascular and mesophyll regions. For each inoculated leaf, after excising the diseased part, if present on specific day, the green regions from the clipped end were cut in transverse orientation as proximal, middle and distal region maintaining 1cm width for each region along the mid-rib. From each region, multiple thin transverse sections (including both vascular and surrounding mesophyll regions) were hand-prepared with a razor blade with each sections approximately 100 to 150 μm thickness. Multiple sections from different parts of the infected leaves were then mounted on separate glass slides (Rohem Industries pvt. Ltd; IS-3099) and directly scanned under CLSM (LSM700; Carl Zeiss, Germany) for the bacterial cells with green and red fluorescence separately indicating the presence of the *gfp* (excitation: 488 nm and emissions: 505 to 550 nm band pass, with 518 nm emission maximum) and *rfp* (excitation: 555 nm and emissions: 582 to 800 nm band pass, with 585 nm emission maximum) marker genes. Multiple *Z* section scans were acquired at 0.5 μm increments for large aggregates in each field. The aggregate size was determined by dividing the area of an aggregate within a single *Z* section by the area of a single cell, followed by addition of results of all the *Z* sections spanning the size of the entire aggregate for wild-type bioreporter strains of *Xcc*. At least three sections were sampled from proximal, middle and distal regions of each leaf with the experimental repeat for thrice independently.

### Histochemical staining of the inoculated cabbage leaves for *in planta* GUS activity

The cabbage leaves inoculated with wild-type GUS reporter strain *Xcc* 8004 (pLAFR6/*P*_*eng*_:*gusA*) were harvested on dpi 1, 3, 6, 9 and 12 and were stained with 1 mM of chromogenic substrate X-Gluc (5-bromo-4-chloro-3-indolyl-β-D-glucuronide) in GUS assay buffer [50 mM sodium di-hydrogen phosphate (pH 7.0), 10 mM EDTA, 0.1% sodium lauryl sarcosine, 0.1% Triton X-100 and 10 mM β-mercaptoethanol] to determine *in planta* β-glucuronidase activity. Briefly, each leaf was subjected to vacuum (15 psi) application for 1 hr to facilitate X-Gluc penetration into the infiltrated leaves and then incubated at 37°C for 2 hr [32, 35]. Subsequently, chlorophyll was completely removed from the stained leaves by incubating in absolute ethanol for 72 hr at 37°C followed by observation under white light by using a bright field stereomicroscope (SteREO Lumar. V12; Carl Zeiss) for different blue coloured stained regions for the *in planta* GUS activity. The experiment was performed with a minimum of five infected leaves per plant for total 5 plants and repeated thrice.

### CFU assay for *in planta* bacterial growth

CFU assay was performed on the nutrient rich solid PSA plates supplemented with suitable antibiotics to calculate the *in planta* growth of different bioreporter strains of *Xcc* 8004. On specific dpi(s), bacterial CFUs were obtained for 1 cm^2^ green leaf region proximal to the clip inoculation site from surface sterilized cabbage leaves. The leaves were surface sterilized by dipping in 1% (vol/vol) sodium hypochlorite for 2 min followed by three washes with sterile MQ water and then crushed with 1 ml of autoclaved MQ water using sterile mortar and pestle, and further dilution plated at concentrations. After sufficient incubation of the inoculated plates at 28°C, the no. of optimally developed bacterial colonies for each combination were observed for their constitutive *gfp* and *rfp* fluorescence under stereomicroscope (SteREO Lumar. V12; Carl Zeiss) and finally normalized to CFUs per cm^2^ proximal leaf region.

### Image analysis and Statistical validation

All the *in vitro* and *in vivo* CLSM raw images were analyzed using ZEN lite 2012 (Carl Zeiss) software for fluorescence pixel intensity calculation minimizing the background intensity, and FIJI (Image J) software for co-localization and final picture brightness correction respectively. The GFP and RFP fluorescence pixel intensities for bacterial cells/populations were represented in Arbitrary Units (A.U.). Statistical comparisons were computed using the Student’s test (non-parametric, paired, two-tailed test) as denoted in figure legends (Prism 5, GraphPad Software). A “p value” of less than 0.05 was considered significant.

## Supporting information

S1 Fig Construction of DSF responsive dual-bioreporter strain of *Xcc*. **(A)** Schematic representation of the construction of DSF responsive dual-bioreporter strain of *Xcc*. *gfp*; green fluorescence protein gene, *rfp*; red fluorescence protein gene. F.P; Forward Primer, R.P; Reverse Primer. MCS; Multiple Cloning Site. Amp^R^; Ampicillin resistance. **(B)** Quantification of average GFP (*P*_*kan*_:*gfp*) and RFP (*P*_*eng*_:*rfp*) fluorescence pixel intensities per bacterial cell in the dual-bioreporter populations of wild-type *Xcc* 8004, along with its Δ*rpfF* (i.e. DSF synthase mutant; as a QS negative control) and *Xcc* Δ*rpfF* supplemented with 4.84 μM DSF at 24 hr of growth. Confocal Laser Scanning Microscopy (CLSM) images were analysed (using ZEN software) for the above quantification, where data analysis was performed by taking six different CLSM images as samples for each strain with at least three experimental repeats and represented with Mean ± SD. P-value for significant difference level was determined by performing student’s T-test (two tailed, paired).
(TIF)

S2 Fig *Xcc* exhibits QS heterogeneity temporally in response to DSF at high cell density *in vitro*. **(A)** Representative confocal images depicting *gfp* and *rfp* expression of dual-bioreporter cells of wild-type *Xcc* 8004, along with *Xcc* Δ*rpfF* (as a QS negative control) and *Xcc* Δ*rpfF* (supplemented with 4.84μM external DSF) at optimal (i.e. 24 hours) growth in liquid PS media (from top to bottom). The panels for each strain (left to right) show *gfp*, *rfp* and their merged images respectively. Images were prepared using FIJI (image J) software. Scale bars on each panel, 5 μm. **(B)** Quorum induction dynamics within the bioreporter populations of *Xcc* 8004 and *Xcc* Δ*rpfF* (supplemented with 4.84μM external DSF); showing the percent of QS-induced cells and quorum induced red fluorescent protein (RFP) pixel intensity per cell at different bacterial cell densities (log CFU/ml). Data analysis was performed (using ZEN software) by taking six different confocal images as samples for each strain at a time for QS induction calculation, with the experimental repeats of at least thrice and represented with Mean ± SD for each time point.
(TIF)

S3 Fig *Xcc* experiences DSF responsive heterogeneous QS-response temporally at a high cell density *in vitro*. Representative CLSM images for constitutive *gfp* and DSF responsive *rfp* expression fluorescence dynamics in liquid PS media for whole-cell QS dual-bioreporter strains of **(A)** wild-type *Xcc* 8004, and **(B)** its DSF deficient Δ*rpfF* mutant supplemented initially with 4.84μM external DSF. The panels for each strain (left to right) show representative *gfp*, *rfp* and their merged images of each specified sampling time upto 44 hr of growth (from top to bottom) respectively. Images were prepared using LSM image browser software. Scale bars on each panel, 10 μm.
(TIF)

S4 Fig DSF deficient mutant (*ΔrpfF*) of *Xcc* exhibit basal level promoter expression (*P*_*eng*_:*rfp*) *in vitro*. **(A)** Representative CLSM images for constitutive *gfp* and DSF responsive *rfp* expression fluorescence dynamics in liquid PS media for DSF deficient *Xcc* Δ*rpfF* dual-bioreporter at different stages of growth upto 44 hrs after inoculation (from top to bottom). The panels for each strain (left to right) show *gfp*, *rfp* and their merged images respectively. Images were prepared using LSM image browser software. Scale bars on each panel, 10 μm. **(B)** Quantification of background RFP pixel intensity dynamics per bacterial cell within the DSF deficient *Xcc* Δ*rpfF* population (as QS negative control) at different cell densities (log CFU/ml) for basal level DSF responsive promoter expression. Data analysis was performed (using ZEN software) by taking six different confocal images as samples for each strain at a time with the experimental repeat of at least thrice and represented with Mean ± SD.
(TIF)

S5 Fig QS heterogeneity exhibits a bi-modal distribution within *Xcc* population at high cell density *in vitro*. CLSM analysis of 100 representative bacterial cells for their constitutive *gfp* and QS-responsive *rfp* expression patterns within the dual-bioreporter populations of **(A)** wild-type *Xcc*, **(B)** *Xcc* Δ*rpfF* (as a QS negative control) and **(C)** *Xcc* Δ*rpfF* (supplemented with 4.84μM external DSF) at different stages of growth *in vitro*. The panels for each strain (top to bottom) represent QS distribution within the population at 0 hr, 24 hr and 36 hr of growth respectively. Each diamond symbol represents a single bacterial bioreporter cell observed under CLSM. Red diamonds; QS-induced bioreporter cells (cells expressing both *gfp* and *rfp*), Green diamonds; QS uninduced bioreporter cells (cells expressing only *gfp*). % I; Percent QS-induced population, % U; Percent QS uninduced population. **(D)** Quantification of the average QS-responsive RFP pixel Intensity (A.I.) within the bacterial populations during different growth stages *in vitro*. At each time point, data analysis was performed (using ZEN software) by taking four different confocal images as samples for each strain at a time with the experimental repeat of at least thrice and represented as Mean ± SD; where, both GFP and RFP fluorescence pixel intensities were represented in Arbitrary Units (A.U.).
(TIF)

S6 Fig Colonization dynamics of *Xcc* within proximal region from site of infection in inoculated cabbage leaves. CLSM analysis of different regions of the inoculated cabbage leaves, indicating **(A)** Comparison of overall *in planta* growth efficiency between *Xcc* 8004 dual-bioreporter population and normal wild-type *Xcc* 8004 control population upto dpi 12. **(B)** Spatio-temporal localization of the *Xcc* 8004 dual-bioreporter population within vascular and mesophyll regions of the infected cabbage leaves upto dpi 12; where the dotted red box indicates the initial dpi(s) with maximum growth rate of *Xcc* 8004 dual-bioreporter populations within proximal vascular regions as compared to within surrounding mesophyll regions. The day of plant infection was considered as dpi 0. For *inplanta Xcc* 8004 dual-bioreporter population, the bacterial no. and fluorescence pixel intensities were calculated using ZEN software; whereas the *inplanta* population of normal *Xcc* 8004 control, the bacterial no. was calculated from their DIC images with proper adjustment using the ZEN software also. The bacterial population size observed was normalized; values are expressed per cm^2^ leaf region. The characteristics of the total region of the leaf observed on each sampling day were slightly different. Data analysis was done by taking six different confocal images as samples for each strain at a time with the experimental repeat of at least thrice and represented with Mean ± SD. P-values for significant difference level were determined by performing student’s T-test (two tailed, paired). ***; p < 0.005, **; p < 0.05.
(TIF)

S7 Fig *In planta* spatio-temporal localization pattern of QS-responsive *gus* and *gfp* reporter strains of *Xcc*. 40 days old healthy cabbage leaves were clip inoculated with DSF responsive *gus* and *gfp* reporter strains of *Xcc* 8004 separately at a approximate cell density of 10^7^ cells/ml of culture at which QS induction has yet to be occur, and the *gus* and *gfp* reporter gene expression under the control of *P*_*eng*_ (*Xanthomonas endoglucanase* gene; XC_0639) promoter within the bacterial populations along with their *in planta* localization patterns were monitored upto dpi 12 within the infected leaves. **(A)** Histochemical staining for β-glucuronidase activity in cabbage leaves clipped with DSF responsive *gus* reporter strain *Xcc* (pLAFR6/*P*_*eng*_:*gusA*). The panels (from left to right) represent the QS-induced bacterial localization dynamics on dpi(s) 1, 3, 6, 9 and 12 under bright field with stereomicroscope. Blue coloured region; QS-induced β-glucuronidase activity of bacterial population. Yellow arrows on each panel indicate the GUS (blue coloured) staining within vein and their surrounding mesophyll regions in the infected leaves. Scale bars for each panel, 1 cm. **(B)** *In planta* localization of QS-induced wild-type *Xcc* 8004 biosensor cells harbouring the DSF responsive *gfp* reporter gene (pKLN55/*P*_*eng*_:*gfp*) within transverse sections of proximal green regions (1 cm distance from the inoculation site) on dpi 9 spanning **(i)** xylem vessels, **(ii)** guard cells surrounding the xylem vessels in the vascular region and **(iii)** the mesophyll parenchyma (with chloroplasts) around the vascular region. Bottom panel in (ii) represent the magnified images of the white boxed inset of its top panel. The panels for left to right show *gfp*, *rfp*, *gfp*-*rfp* merged, DIC, *gfp*-*rfp*-DIC merged CLSM images respectively. Significant RFP fluorescence in second panels (from left to right) for each site is mainly due to chloroplasts auto-fluorescence. Bacterial localization and fluorescence were analysed using FIJI (image J) and ZEN softwares. Images were prepared using FIJI (image J) software. Data analysis was done by taking six different confocal images from three different infected cabbage leaves as samples for each strain at a time with the experimental repeat of at least thrice.
(TIF)

S8 Fig *gfp* and *mCherry* expression constitutively *in planta* does not alter the survival fitness of *Xcc*. Preliminary *in planta* competition assay, indicating similar levels of survival fitness of wild-type *Xcc* 8004 (QS responders) inspite of expressing either constitutive *P*_*kan*_:*gfp* or *P*_*lac*_:*mCherry* respectively in single and co-cultures (in 1:1 ratio, from ~ 10^7^ cells ml^-1^ 2^o^ culture) upto dpi 12. **(A)** Representative dpi 6 CLSM pictures indicating the presence of individual QS-responding *Xcc* populations within the same transverse section of proximal green vascular regions (upto 1 cm distance from the clipped site excluding diseased part) of cabbage leaf co-inoculated with of *Xcc* 8004/*P*_*kan*_:*gfp* and *Xcc* 8004/*P*_*lac*_:*mCherry* bioreporter cells. The panels from left to right show the *gfp*, *mCherry*, *gfp-mCherry* merged, DIC and *gfp-mCherry-*DIC merged images respectively. Images were prepared using FIJI (image J) software. Scale bars on each panel, 5 μm. **(B)** *In planta* growth of bacterial populations for single and mixed inoculations. **(C)** *In planta* percentage survival of bacterial populations for mixed infection. WT; wild-type *Xcc* 8004. The bacterial population size observed was normalized; values are expressed per cm^2^ leaf region. The characteristics of the total region of the leaf observed on each sampling day were slightly different. Bacterial fluorescence and quantification were analysed using FIJI (image J) software. Data analysis was done by taking six different sites from three infected leaves as samples for each combination at a time with the experimental repeat of at least thrice and represented with Mean ± SD. P-values for significant difference level were determined by performing student’s T-test (two tailed, paired). n.s.; not significant.
(TIF)

S9 Fig QS responders restrict the fitness of QS mutants in *Xcc* during late stage of infection. *In planta* competition assay indicating a significantly compromised survival fitness of QS mutants (QS^-^) in presence of QS responders (QS^+^) of *Xcc* at late stage of infection, where **(A)** Representative CLSM pictures for **(i)** Single infection (with the panels of merged images of green and red fluorescence), and **(ii)** Mixed infections (i.e. co-inoculation; with the panels of *mCherry*, *gfp* and *gfp-mCherry* merged images from left to right respectively); indicating the cell densities of QS^+^ and QS^-^ cells of *Xcc* 8004/*mCherry*, *Xcc* Δ*rpfF*/*gfp* and *Xcc* Δ*rpfC*/*gfp* bioreporter strains spanning transverse sections of leaf vascular regions on dpi 1, 6 and 12 (from top to bottom). Images were prepared using FIJI (image J) software. Scale bars on each panel, 5 μm. **(B)** Quantification of QS^+^ and QS^-^ bacterial populations for single and mixed infections per cm^2^ proximal regions of inoculated leaves on dpi 1, 6 and 12. **(C)** Quantification of percentage survival of QS^+^ and QS^-^ bacterial populations for mixed infection per cm^2^ proximal region of inoculated leaves on dpi 1, 6 and 12. WT; wild-type *Xcc* 8004, Δ*F*; *Xcc* Δ*rpfF*, and Δ*C*; *Xcc* Δ*rpfC*. The bacterial population size observed was normalized; values are expressed per cm^2^ leaf region. Bacterial fluorescence and quantification were analysed from CLSM images using FIJI (image J) software. The characteristics of the total region of the leaf observed on each sampling day were slightly different. Data analysis was done by taking six different sites from three infected leaves as samples for each combination at a time with the experimental repeat of at least thrice and represented with Mean ± SD. P-values for significant difference level were determined by performing student’s T-test (two tailed, paired). ***; p < 0.001.
(TIF)

S10 Fig CFU analysis re-confirms compromised fitness of QS mutants in *Xcc* population during late infection stage. Bacterial CFU analysis for the *in planta* competition assay, indicating the growth and survibility patterns for bacterial population of single bioreporter strains of wild-type *Xcc* 8004 expressing constitutive *gfp*, *Xcc* Δ*rpfF* expressing constitutive *mCherry*, and *Xcc* Δ*rpfC* expressing constitutive *mCherry* within the inoculated cabbage leaves upto dpi 12. **(A)** Total bacterial CFUs (in log scale) for *in planta* bacterial populations of single bioreporter for single and mixed inoculations, and **(B)** percentage survival of wild-type and DSF synthesis mutant bacterial populations for mixed infection. The total no. of bacterial CFUs observed for each bioreporter strain was normalized; values are expressed per cm^2^ proximal leaf region. Data analysis was done by taking six different sites from three infected leaves as samples for each combination at a time with the experimental repeat of at least thrice and represented with Mean ± SD. P-values for significant difference level were determined by performing student’s T-test (two tailed, paired). ***; p < 0.001.
(TIF)

S11 Fig QS-response maximizes the *in planta* migration rate in *Xcc* for their better survibility. Frequency distribution of QS^+^ and QS^-^ bacterial populations in the *in planta* competition assay, indicating the growth and migration patterns for QS responder (i.e. *Xcc* 8004/*mCherry*), QS null (i.e. *Xcc* Δ*rpfF*/*gfp*) and QS blind (i.e. *Xcc* Δ*rpfC*/*gfp*) cells for single as well as mixed infections within clip inoculated cabbage leaves under a CLSM upto dpi 12. **(A)** Average bacterial population size per 1 cm^2^ leaf regions, and **(B)** Spatio-temporal distribution of bacterial population size per 1 cm^2^ proximal, middle and distal leaf regions respectively, for single and mixed infections on dpi 1, 6 and 12. WT; wild-type *Xcc* 8004, Δ*F*; *Xcc* Δ*rpfF*, and Δ*C*; *Xcc* Δ*rpfC*. On specified sampling dpi, multiple Z-stalks were acquired under a CLSM for each sample under green and red fluorescence along with DIC channel, maintaining 0.5 μm gap between two successive Z-planes. Bacterial population size was analysed by considering the X,Y and Z planes for each Z-stalk, where the bacterial cells present in all the Z-planes were counted manually and summed up to calculate the total no. of bacterial cells in that region at a time. The total population size observed was normalized; values are expressed per cm^2^ leaf region. The bacterial population size for each infection was determined by combining the analysed data for five sites per inoculated leaf, six leaves on each sampling day with experimental repeats for thrice. The characteristics of the total region of the leaf observed on each sampling day were slightly different. Data analysis [using FIJI (image J) software] was performed by taking six different confocal images as samples for each strain at a time with the experimental repeat of at least thrice and represented with Mean ± SD. P-values for significant difference level were determined by performing student’s T-test (two tailed, paired). ***; p < 0.001.
(TIF)

S12 Fig Reduced cell-aggregation in QS mutants cannot be rescued by QS responders of *Xcc in planta*. Cell aggregate formation patterns of QS responder (i.e. *Xcc* 8004/*mCherry*), QS null (i.e. *Xcc* Δ*rpfF*/*gfp*) and QS blind (i.e. *Xcc* Δ*rpfC*/*gfp*) cells for single as well as mixed infections in the *in planta* competition assay within clip inoculated cabbage leaves under a CLSM upto dpi 12. **(A)** Average no. of bacterial aggregates per 1 cm^2^ leaf regions, and **(B)** Spatio-temporal distribution of no. of bacterial aggregates per 1 cm^2^ proximal, middle and distal leaf regions respectively, for single and mixed infections on dpi 1, 6 and 12. WT; wild-type *Xcc* 8004, Δ*F*; *Xcc* Δ*rpfF*, and Δ*C*; *Xcc* Δ*rpfC*. On specified sampling dpi, multiple Z-stalks were acquired under a CLSM for each sample under green and red fluorescence along with DIC channel, maintaining 0.5 μm gap between two successive Z-planes. Bacterial aggregate size as well as no. were analysed by considering the X,Y and Z planes for each aggregate of a Z-stalk, where the bacterial aggregates present in all the Z-planes were counted manually and summed up to calculate their total no. in that region at a time. Total bacterial aggregate no. observed was normalized and the values are expressed per cm^2^ leaf region. The no. of bacterial aggregates for each infection was determined by combining the analysed data for five sites per inoculated leaf, six leaves on each sampling day with experimental repeats for thrice. The characteristics of the total region of the leaf observed at each sampling time were slightly different. Data analysis [using FIJI (image J) software] was performed by taking six different confocal images as samples for each strain at a time with the experimental repeat of at least thrice and represented with Mean ± SD. P-values for significant difference level were determined by performing student’s T-test (two tailed, paired). ***; p < 0.001.
(TIF)

S13 Fig Frequency distribution of bacterial aggregates of *Xcc* populations *in planta*. Frequency distribution of bacterial aggregates of *Xcc 8004*/*mCherry* (WT), *Xcc* Δ*rpfF*/*gfp* (DSF synthesis mutant) and *Xcc* Δ*rpfC*/*gfp* (DSF sensor mutant) bioreporter populations within inoculated cabbage leaves on dpi 1, 6 and 12 for individual as well as mixed (co-inoculated with 1:1 ratios, from ~ 10^7^ cells ml^-1^ 2^o^ culture) infections. **(A)** Single Infection studies; with (i) *Xcc 8004*/*mCherry* (WT), (ii) *Xcc* Δ*rpfF*/*gfp* (DSF synthesis mutant) and (iii) *Xcc* Δ*rpfC*/*gfp* (DSF sensor mutant), **(B)** Mixed Infection studies; with (i) *Xcc 8004*/*mCherry* (WT) vs. (ii) *Xcc* Δ*rpfF*/*gfp* (DSF synthesis mutant), and **(C)** Mixed Infection studies; with (i) *Xcc 8004*/*mCherry* (WT) vs. (ii) *Xcc* Δ*rpfC*/*gfp* (DSF sensor mutant). On specific dpi, the bacterial aggregates were observed and their frequency distribution were calculated within proximal, middle and distal green regions to the infection site spanning approximately 1cm distance for each region. The total number of bacterial aggregates for each infection was determined for six leaves at each sampling time, and the data were combined. The characteristics of the total region of the leaf observed at each sampling time were slightly different. The number of cells per aggregate was estimated from the surface area and depth of each aggregate using FIJI (image J) software. The total number of aggregates observed was normalized; values are expressed per cm^2^ leaf region.
(TIF)
