# Supplementary figures and images for: New insight into bacterial social communication in natural host: Evidence for interplay of heterogeneous and unison quorum response
Source: PLoS Genet. 2019 Sep 17;15(9):e1008395. doi: 10.1371/journal.pgen.1008395 (PMC6764700; doi:10.1371/journal.pgen.1008395)

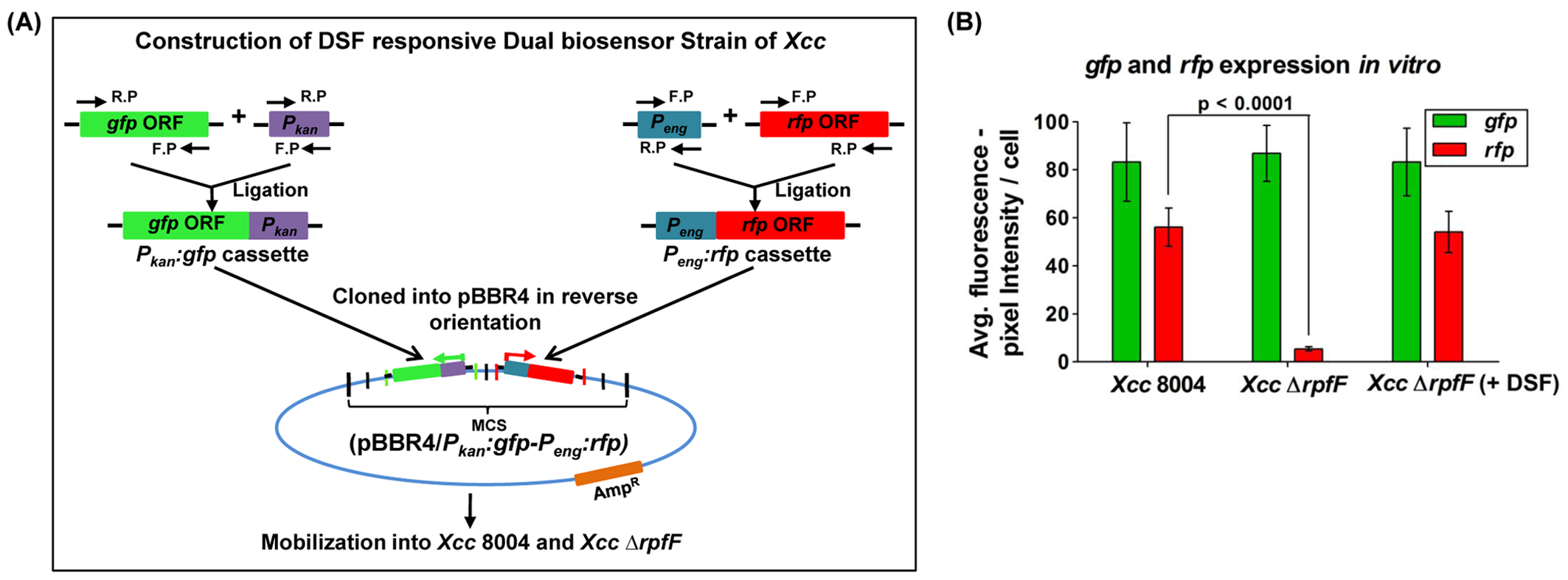

Supplement: S1 Fig — (A) Schematic representation of the construction of DSF responsive dual-bioreporter strain of Xcc. gfp; green fluorescence protein gene, rfp; red fluorescence protein gene. F.P; Forward Primer, R.P; Reverse Primer. MCS; Multiple Cloning Site. AmpR; Ampicillin resistance. (B) Quantification of average GFP (Pkan:gfp) and RFP (Peng:rfp) fluorescence pixel intensities per bacterial cell in the dual-bioreporter populations of wild-type Xcc 8004, along with its ΔrpfF (i.e. DSF synthase mutant; as a QS negative control) and Xcc ΔrpfF supplemented with 4.84 μM DSF at 24 hr of growth. Confocal Laser Scanning Microscopy (CLSM) images were analysed (using ZEN software) for the above quantification, where data analysis was performed by taking six different CLSM images as samples for each strain with at least three experimental repeats and represented with Mean ± SD. P-value for significant difference level was determined by performing student’s T-test (two tailed, paired). (TIF) [file pgen.1008395.s001.tif]

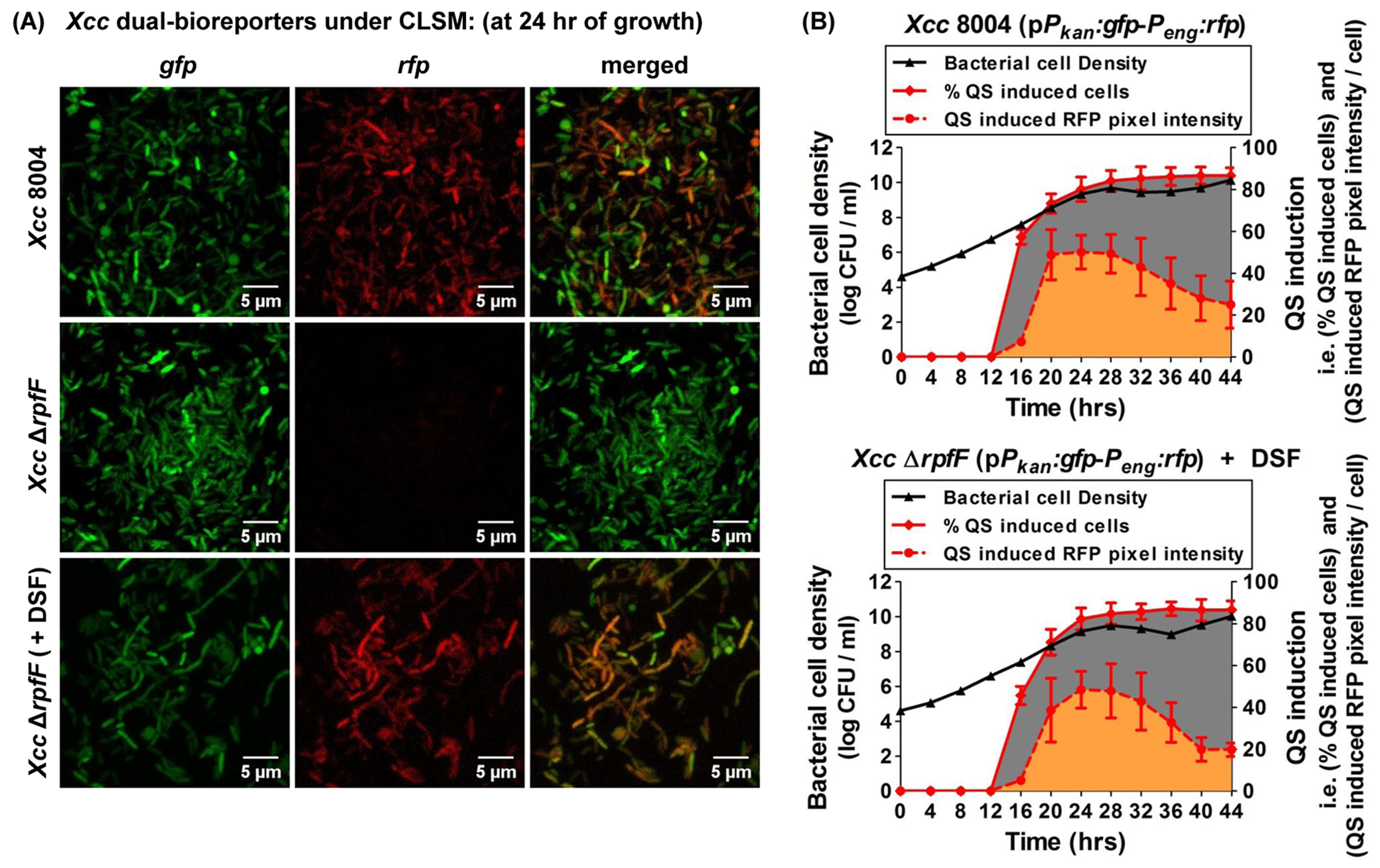

Supplement: S2 Fig — (A) Representative confocal images depicting gfp and rfp expression of dual-bioreporter cells of wild-type Xcc 8004, along with Xcc ΔrpfF (as a QS negative control) and Xcc ΔrpfF (supplemented with 4.84μM external DSF) at optimal (i.e. 24 hours) growth in liquid PS media (from top to bottom). The panels for each strain (left to right) show gfp, rfp and their merged images respectively. Images were prepared using FIJI (image J) software. Scale bars on each panel, 5 μm. (B) Quorum induction dynamics within the bioreporter populations of Xcc 8004 and Xcc ΔrpfF (supplemented with 4.84μM external DSF); showing the percent of QS-induced cells and quorum induced red fluorescent protein (RFP) pixel intensity per cell at different bacterial cell densities (log CFU/ml). Data analysis was performed (using ZEN software) by taking six different confocal images as samples for each strain at a time for QS induction calculation, with the experimental repeats of at least thrice and represented with Mean ± SD for each time point. (TIF) [file pgen.1008395.s002.tif]

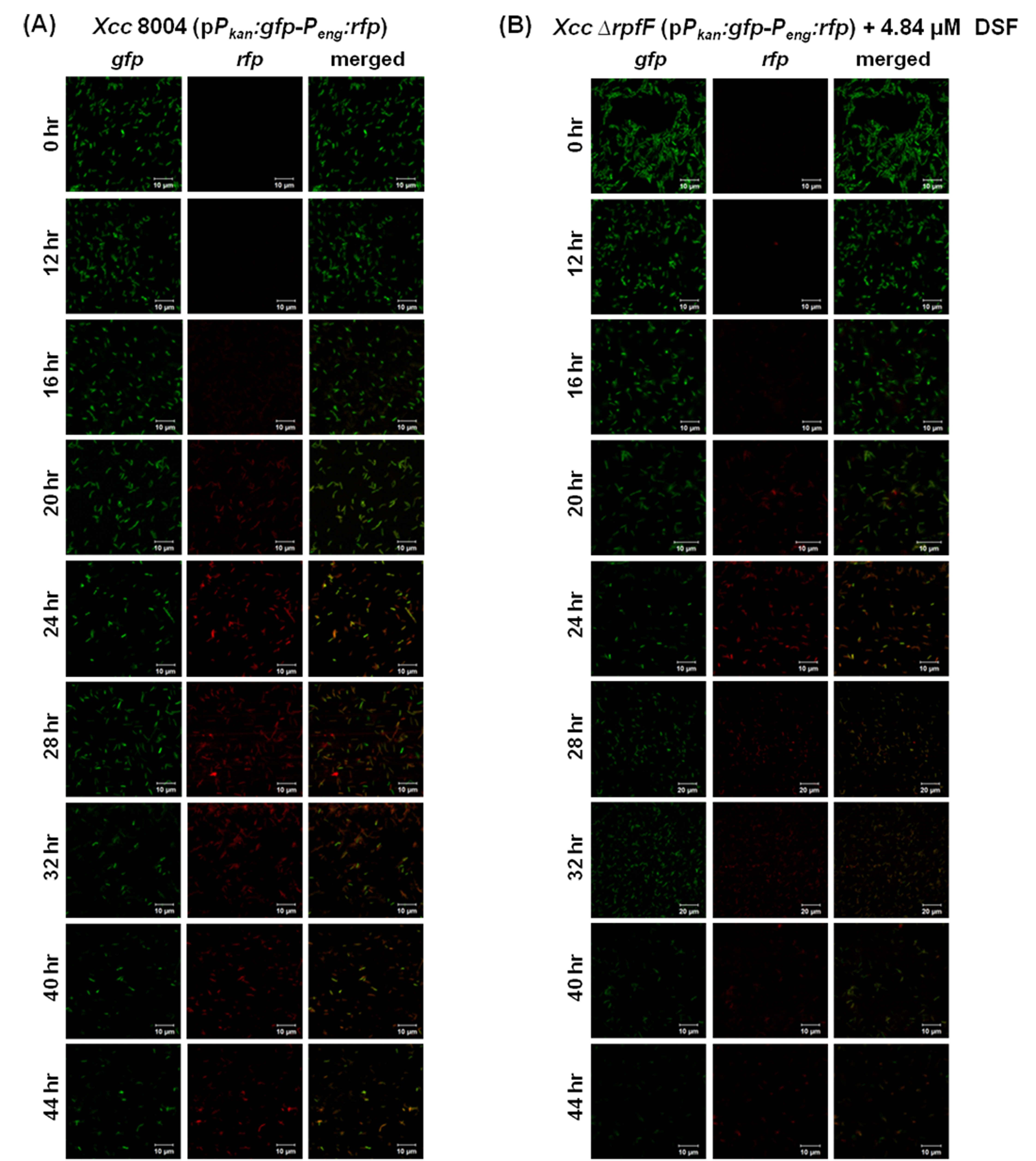

Supplement: S3 Fig — Representative CLSM images for constitutive gfp and DSF responsive rfp expression fluorescence dynamics in liquid PS media for whole-cell QS dual-bioreporter strains of (A) wild-type Xcc 8004, and (B) its DSF deficient ΔrpfF mutant supplemented initially with 4.84μM external DSF. The panels for each strain (left to right) show representative gfp, rfp and their merged images of each specified sampling time upto 44 hr of growth (from top to bottom) respectively. Images were prepared using LSM image browser software. Scale bars on each panel, 10 μm. (TIF) [file pgen.1008395.s003.tif]

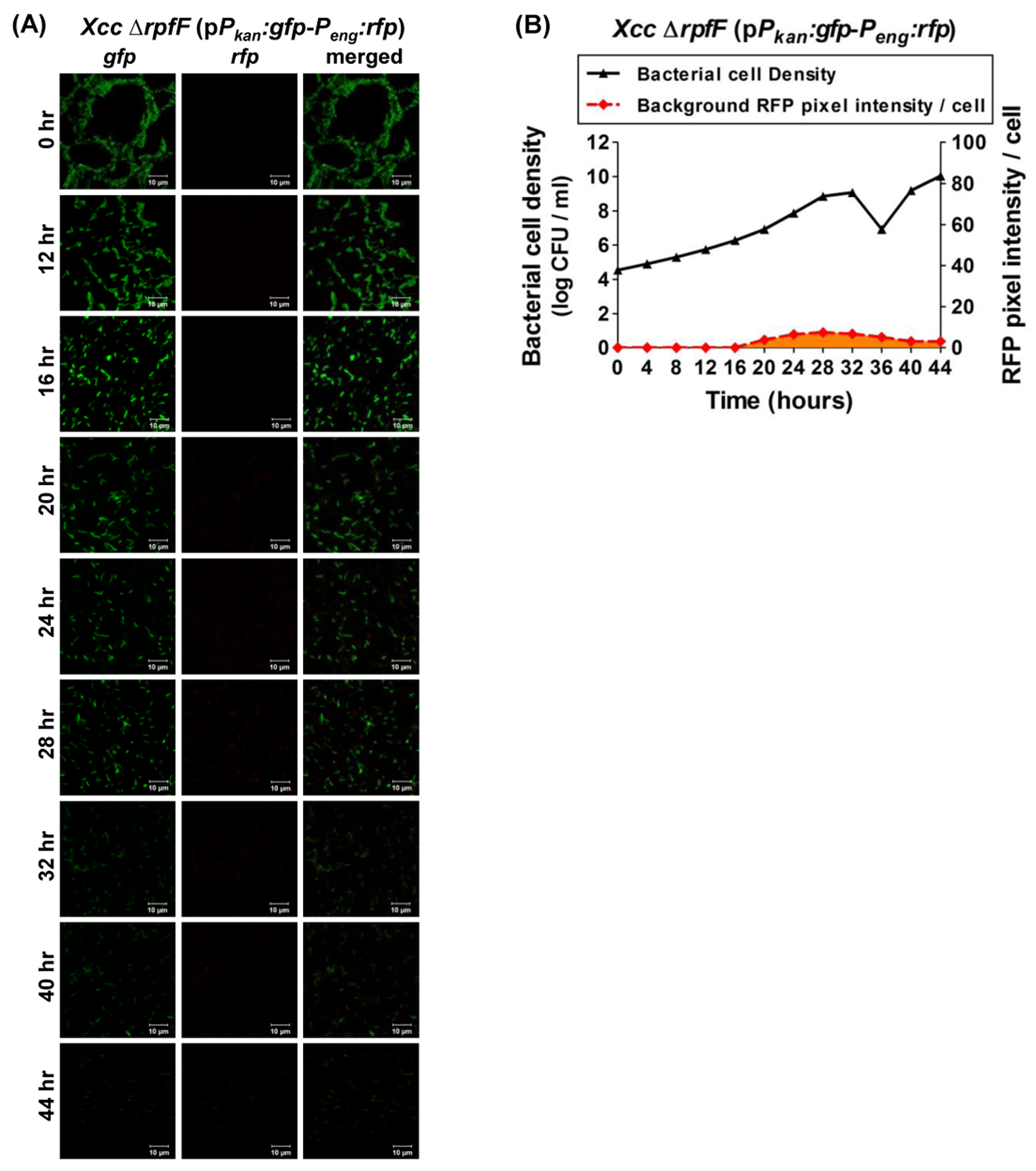

Supplement: S4 Fig — (A) Representative CLSM images for constitutive gfp and DSF responsive rfp expression fluorescence dynamics in liquid PS media for DSF deficient Xcc ΔrpfF dual-bioreporter at different stages of growth upto 44 hrs after inoculation (from top to bottom). The panels for each strain (left to right) show gfp, rfp and their merged images respectively. Images were prepared using LSM image browser software. Scale bars on each panel, 10 μm. (B) Quantification of background RFP pixel intensity dynamics per bacterial cell within the DSF deficient Xcc ΔrpfF population (as QS negative control) at different cell densities (log CFU/ml) for basal level DSF responsive promoter expression. Data analysis was performed (using ZEN software) by taking six different confocal images as samples for each strain at a time with the experimental repeat of at least thrice and represented with Mean ± SD. (TIF) [file pgen.1008395.s004.tif]

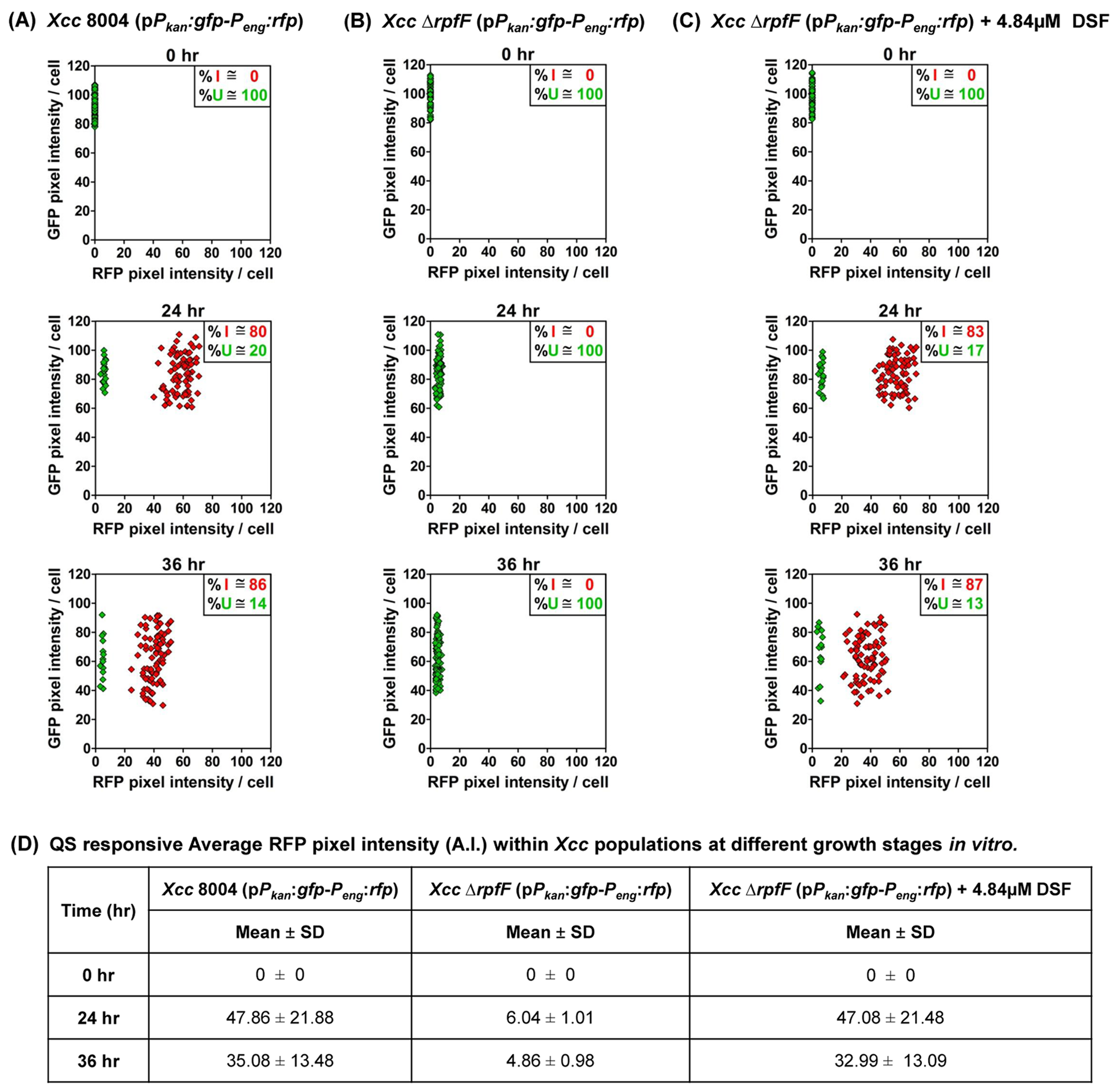

Supplement: S5 Fig — CLSM analysis of 100 representative bacterial cells for their constitutive gfp and QS-responsive rfp expression patterns within the dual-bioreporter populations of (A) wild-type Xcc, (B) Xcc ΔrpfF (as a QS negative control) and (C) Xcc ΔrpfF (supplemented with 4.84μM external DSF) at different stages of growth in vitro. The panels for each strain (top to bottom) represent QS distribution within the population at 0 hr, 24 hr and 36 hr of growth respectively. Each diamond symbol represents a single bacterial bioreporter cell observed under CLSM. Red diamonds; QS-induced bioreporter cells (cells expressing both gfp and rfp), Green diamonds; QS uninduced bioreporter cells (cells expressing only gfp). % I; Percent QS-induced population, % U; Percent QS uninduced population. (D) Quantification of the average QS-responsive RFP pixel Intensity (A.I.) within the bacterial populations during different growth stages in vitro. At each time point, data analysis was performed (using ZEN software) by taking four different confocal images as samples for each strain at a time with the experimental repeat of at least thrice and represented as Mean ± SD; where, both GFP and RFP fluorescence pixel intensities were represented in Arbitrary Units (A.U.). (TIF) [file pgen.1008395.s005.tif]

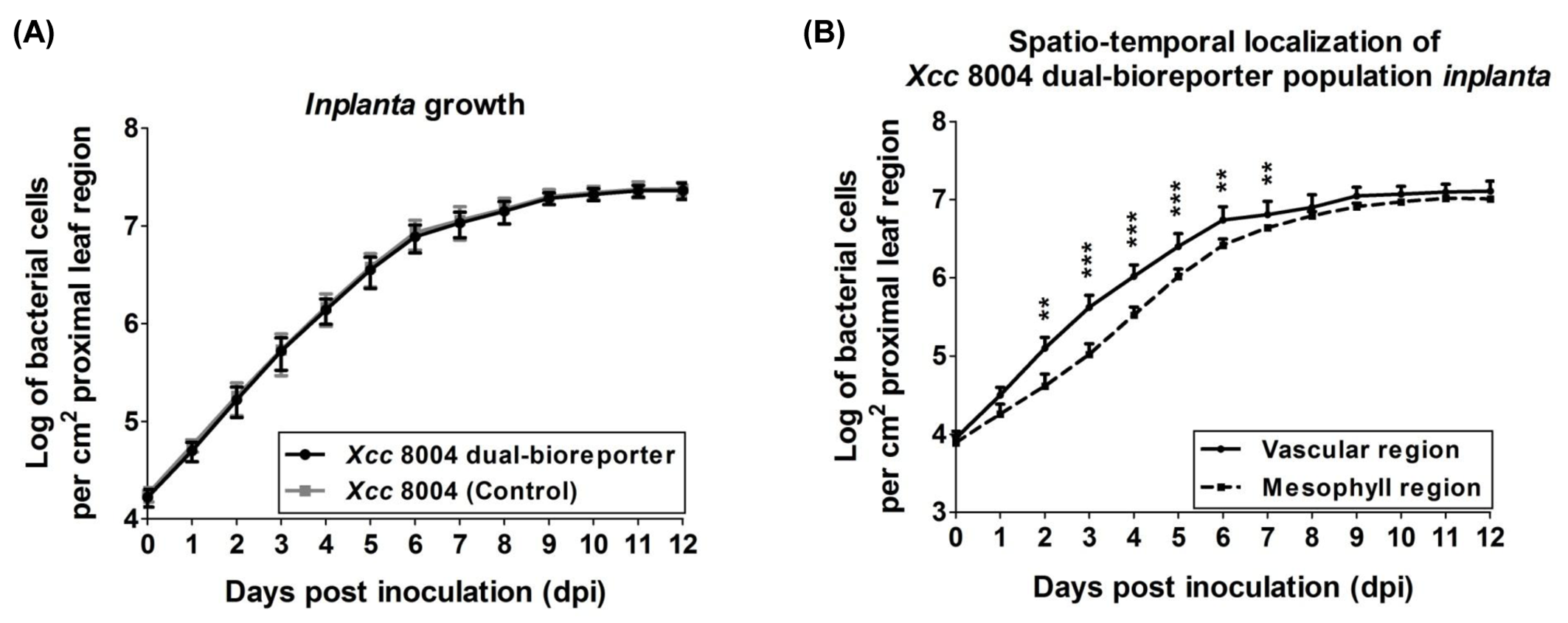

Supplement: S6 Fig — CLSM analysis of different regions of the inoculated cabbage leaves, indicating (A) Comparison of overall in planta growth efficiency between Xcc 8004 dual-bioreporter population and normal wild-type Xcc 8004 control population upto dpi 12. (B) Spatio-temporal localization of the Xcc 8004 dual-bioreporter population within vascular and mesophyll regions of the infected cabbage leaves upto dpi 12; where the dotted red box indicates the initial dpi(s) with maximum growth rate of Xcc 8004 dual-bioreporter populations within proximal vascular regions as compared to within surrounding mesophyll regions. The day of plant infection was considered as dpi 0. For inplanta Xcc 8004 dual-bioreporter population, the bacterial no. and fluorescence pixel intensities were calculated using ZEN software; whereas the inplanta population of normal Xcc 8004 control, the bacterial no. was calculated from their DIC images with proper adjustment using the ZEN software also. The bacterial population size observed was normalized; values are expressed per cm2 leaf region. The characteristics of the total region of the leaf observed on each sampling day were slightly different. Data analysis was done by taking six different confocal images as samples for each strain at a time with the experimental repeat of at least thrice and represented with Mean ± SD. P-values for significant difference level were determined by performing student’s T-test (two tailed, paired). ***; p < 0.005, **; p < 0.05. (TIF) [file pgen.1008395.s006.tif]

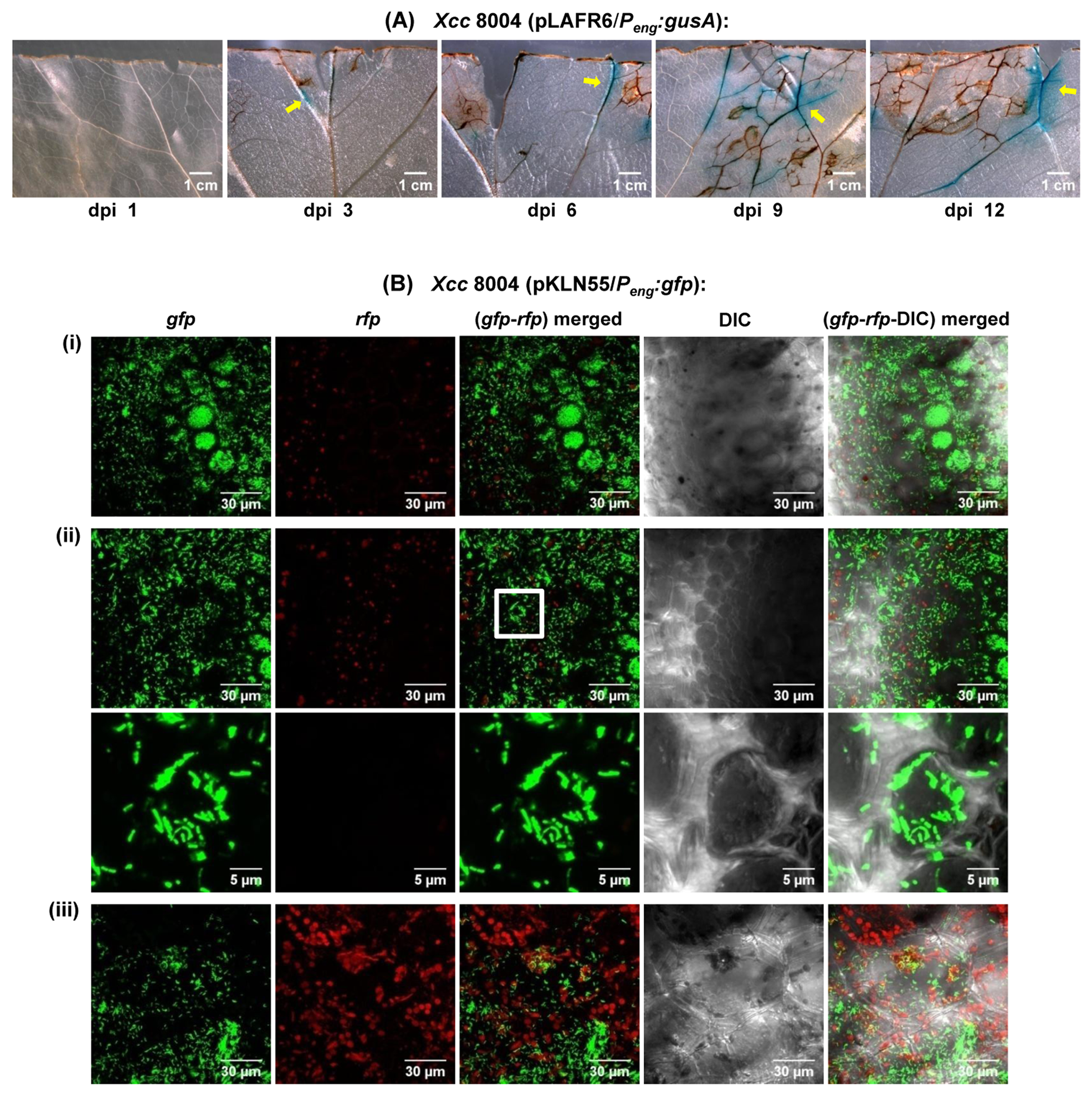

Supplement: S7 Fig — 40 days old healthy cabbage leaves were clip inoculated with DSF responsive gus and gfp reporter strains of Xcc 8004 separately at a approximate cell density of 107 cells/ml of culture at which QS induction has yet to be occur, and the gus and gfp reporter gene expression under the control of Peng (Xanthomonas endoglucanase gene; XC_0639) promoter within the bacterial populations along with their in planta localization patterns were monitored upto dpi 12 within the infected leaves. (A) Histochemical staining for β-glucuronidase activity in cabbage leaves clipped with DSF responsive gus reporter strain Xcc (pLAFR6/Peng:gusA). The panels (from left to right) represent the QS-induced bacterial localization dynamics on dpi(s) 1, 3, 6, 9 and 12 under bright field with stereomicroscope. Blue coloured region; QS-induced β-glucuronidase activity of bacterial population. Yellow arrows on each panel indicate the GUS (blue coloured) staining within vein and their surrounding mesophyll regions in the infected leaves. Scale bars for each panel, 1 cm. (B) In planta localization of QS-induced wild-type Xcc 8004 biosensor cells harbouring the DSF responsive gfp reporter gene (pKLN55/Peng:gfp) within transverse sections of proximal green regions (1 cm distance from the inoculation site) on dpi 9 spanning (i) xylem vessels, (ii) guard cells surrounding the xylem vessels in the vascular region and (iii) the mesophyll parenchyma (with chloroplasts) around the vascular region. Bottom panel in (ii) represent the magnified images of the white boxed inset of its top panel. The panels for left to right show gfp, rfp, gfp-rfp merged, DIC, gfp-rfp-DIC merged CLSM images respectively. Significant RFP fluorescence in second panels (from left to right) for each site is mainly due to chloroplasts auto-fluorescence. Bacterial localization and fluorescence were analysed using FIJI (image J) and ZEN softwares. Images were prepared using FIJI (image J) software. Data analysis was done by taking six d [file pgen.1008395.s007.tif]

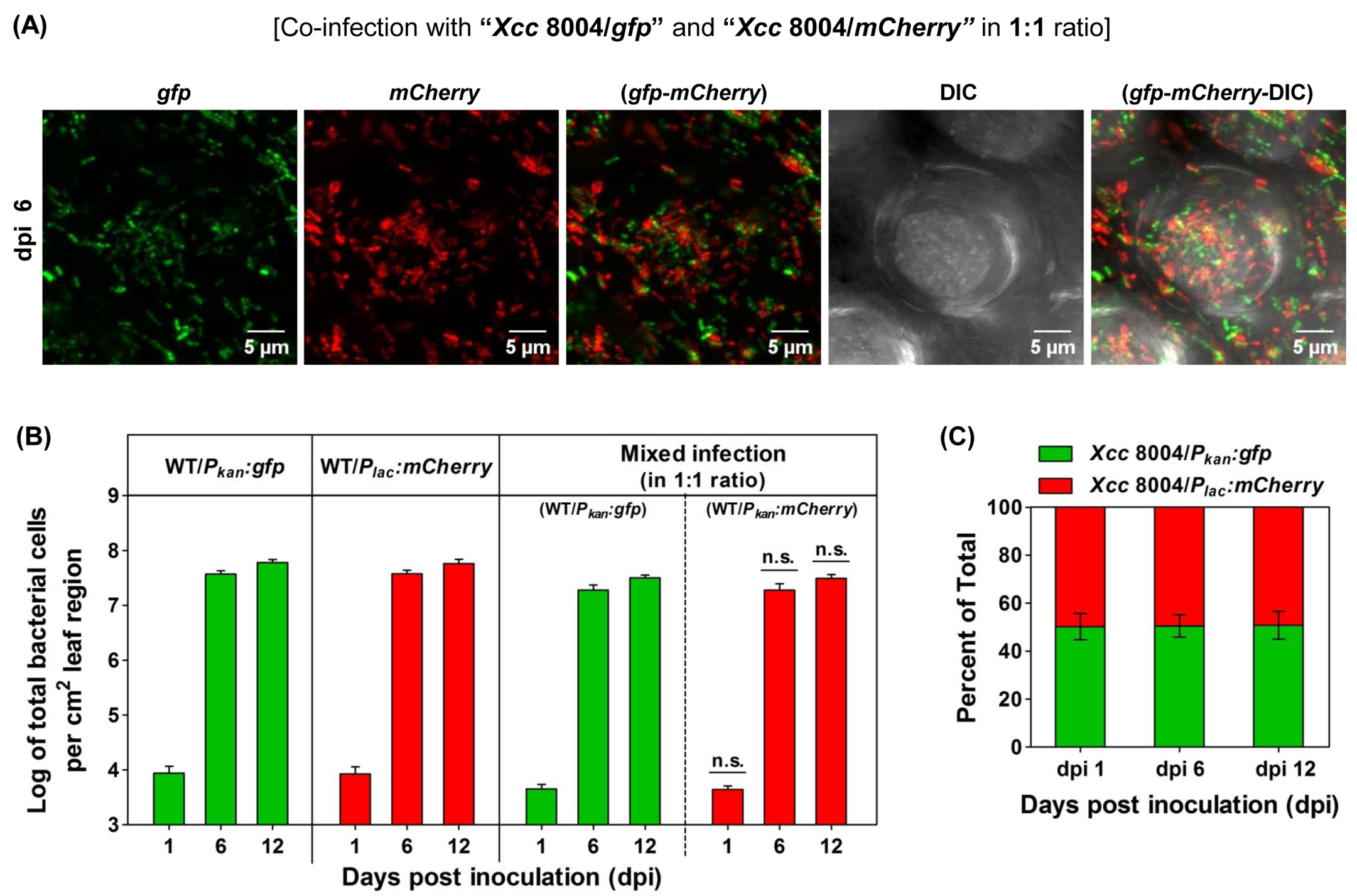

Supplement: S8 Fig — Preliminary in planta competition assay, indicating similar levels of survival fitness of wild-type Xcc 8004 (QS responders) inspite of expressing either constitutive Pkan:gfp or Plac:mCherry respectively in single and co-cultures (in 1:1 ratio, from ~ 107 cells ml-1 2o culture) upto dpi 12. (A) Representative dpi 6 CLSM pictures indicating the presence of individual QS-responding Xcc populations within the same transverse section of proximal green vascular regions (upto 1 cm distance from the clipped site excluding diseased part) of cabbage leaf co-inoculated with of Xcc 8004/Pkan:gfp and Xcc 8004/Plac:mCherry bioreporter cells. The panels from left to right show the gfp, mCherry, gfp-mCherry merged, DIC and gfp-mCherry-DIC merged images respectively. Images were prepared using FIJI (image J) software. Scale bars on each panel, 5 μm. (B) In planta growth of bacterial populations for single and mixed inoculations. (C) In planta percentage survival of bacterial populations for mixed infection. WT; wild-type Xcc 8004. The bacterial population size observed was normalized; values are expressed per cm2 leaf region. The characteristics of the total region of the leaf observed on each sampling day were slightly different. Bacterial fluorescence and quantification were analysed using FIJI (image J) software. Data analysis was done by taking six different sites from three infected leaves as samples for each combination at a time with the experimental repeat of at least thrice and represented with Mean ± SD. P-values for significant difference level were determined by performing student’s T-test (two tailed, paired). n.s.; not significant. (TIF) [file pgen.1008395.s008.tif]

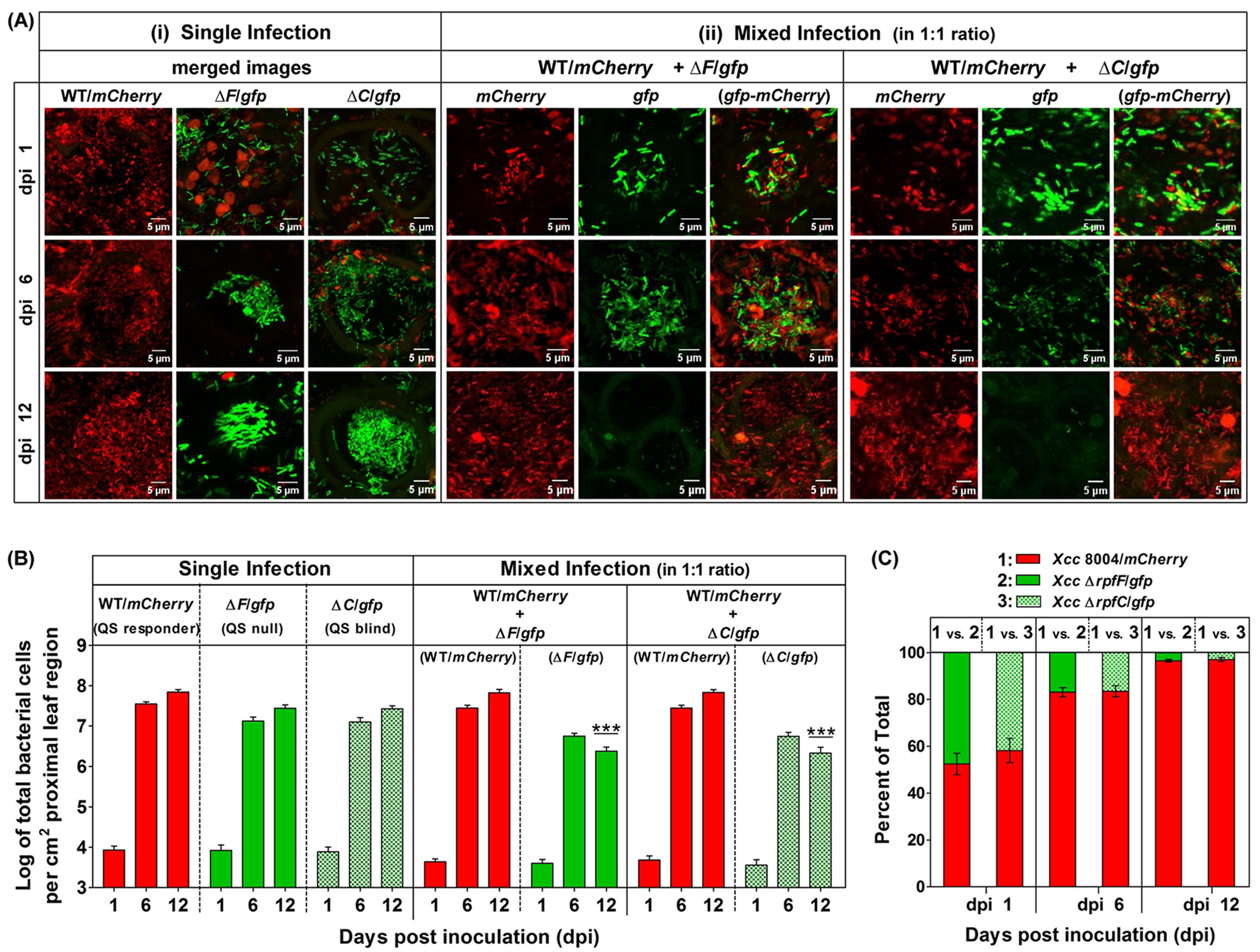

Supplement: S9 Fig — In planta competition assay indicating a significantly compromised survival fitness of QS mutants (QS-) in presence of QS responders (QS+) of Xcc at late stage of infection, where (A) Representative CLSM pictures for (i) Single infection (with the panels of merged images of green and red fluorescence), and (ii) Mixed infections (i.e. co-inoculation; with the panels of mCherry, gfp and gfp-mCherry merged images from left to right respectively); indicating the cell densities of QS+ and QS- cells of Xcc 8004/mCherry, Xcc ΔrpfF/gfp and Xcc ΔrpfC/gfp bioreporter strains spanning transverse sections of leaf vascular regions on dpi 1, 6 and 12 (from top to bottom). Images were prepared using FIJI (image J) software. Scale bars on each panel, 5 μm. (B) Quantification of QS+ and QS- bacterial populations for single and mixed infections per cm2 proximal regions of inoculated leaves on dpi 1, 6 and 12. (C) Quantification of percentage survival of QS+ and QS- bacterial populations for mixed infection per cm2 proximal region of inoculated leaves on dpi 1, 6 and 12. WT; wild-type Xcc 8004, ΔF; Xcc ΔrpfF, and ΔC; Xcc ΔrpfC. The bacterial population size observed was normalized; values are expressed per cm2 leaf region. Bacterial fluorescence and quantification were analysed from CLSM images using FIJI (image J) software. The characteristics of the total region of the leaf observed on each sampling day were slightly different. Data analysis was done by taking six different sites from three infected leaves as samples for each combination at a time with the experimental repeat of at least thrice and represented with Mean ± SD. P-values for significant difference level were determined by performing student’s T-test (two tailed, paired). ***; p < 0.001. (TIF) [file pgen.1008395.s009.tif]

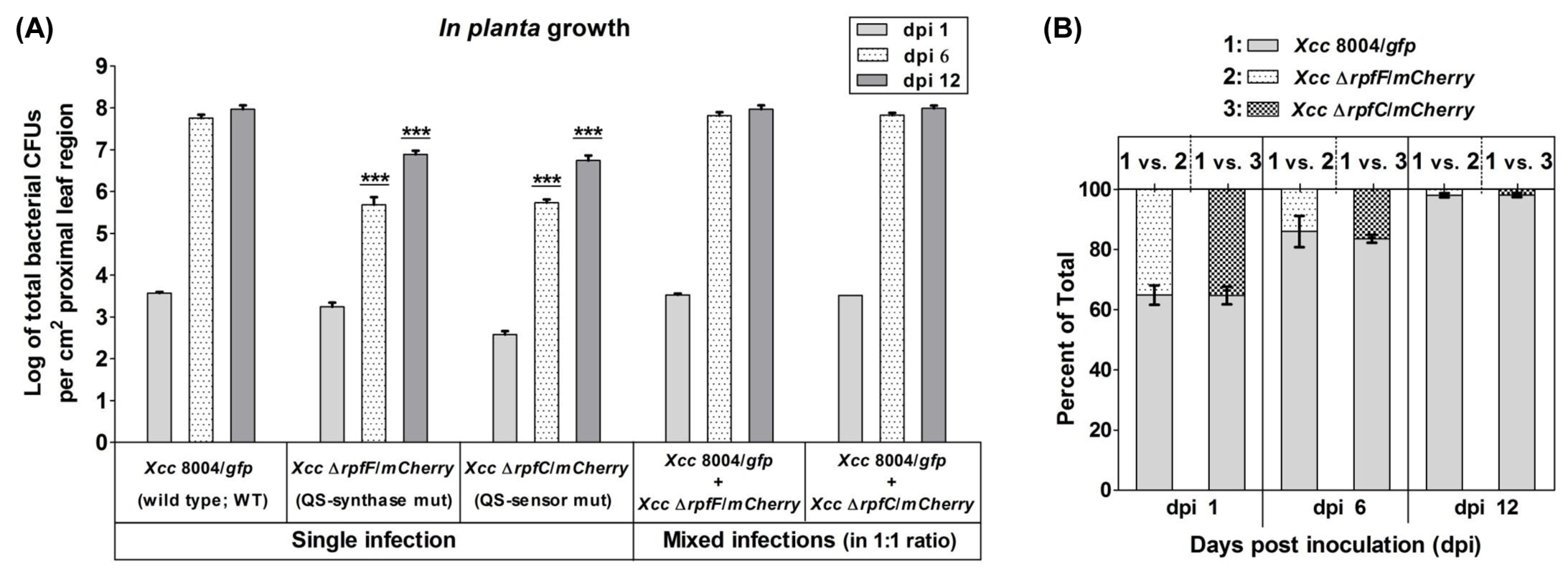

Supplement: S10 Fig — Bacterial CFU analysis for the in planta competition assay, indicating the growth and survibility patterns for bacterial population of single bioreporter strains of wild-type Xcc 8004 expressing constitutive gfp, Xcc ΔrpfF expressing constitutive mCherry, and Xcc ΔrpfC expressing constitutive mCherry within the inoculated cabbage leaves upto dpi 12. (A) Total bacterial CFUs (in log scale) for in planta bacterial populations of single bioreporter for single and mixed inoculations, and (B) percentage survival of wild-type and DSF synthesis mutant bacterial populations for mixed infection. The total no. of bacterial CFUs observed for each bioreporter strain was normalized; values are expressed per cm2 proximal leaf region. Data analysis was done by taking six different sites from three infected leaves as samples for each combination at a time with the experimental repeat of at least thrice and represented with Mean ± SD. P-values for significant difference level were determined by performing student’s T-test (two tailed, paired). ***; p < 0.001. (TIF) [file pgen.1008395.s010.tif]

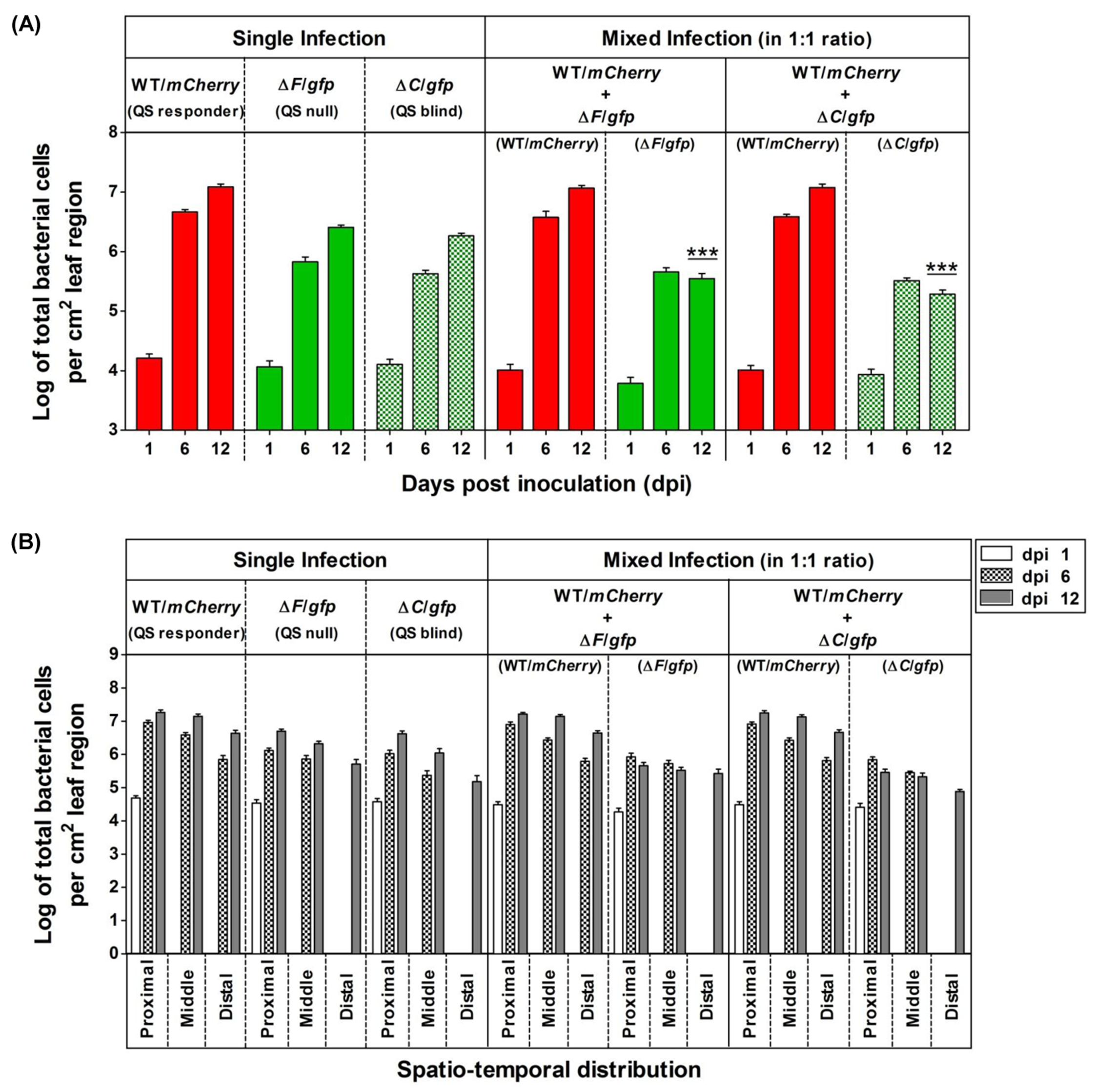

Supplement: S11 Fig — Frequency distribution of QS+ and QS- bacterial populations in the in planta competition assay, indicating the growth and migration patterns for QS responder (i.e. Xcc 8004/mCherry), QS null (i.e. Xcc ΔrpfF/gfp) and QS blind (i.e. Xcc ΔrpfC/gfp) cells for single as well as mixed infections within clip inoculated cabbage leaves under a CLSM upto dpi 12. (A) Average bacterial population size per 1 cm2 leaf regions, and (B) Spatio-temporal distribution of bacterial population size per 1 cm2 proximal, middle and distal leaf regions respectively, for single and mixed infections on dpi 1, 6 and 12. WT; wild-type Xcc 8004, ΔF; Xcc ΔrpfF, and ΔC; Xcc ΔrpfC. On specified sampling dpi, multiple Z-stalks were acquired under a CLSM for each sample under green and red fluorescence along with DIC channel, maintaining 0.5 μm gap between two successive Z-planes. Bacterial population size was analysed by considering the X,Y and Z planes for each Z-stalk, where the bacterial cells present in all the Z-planes were counted manually and summed up to calculate the total no. of bacterial cells in that region at a time. The total population size observed was normalized; values are expressed per cm2 leaf region. The bacterial population size for each infection was determined by combining the analysed data for five sites per inoculated leaf, six leaves on each sampling day with experimental repeats for thrice. The characteristics of the total region of the leaf observed on each sampling day were slightly different. Data analysis [using FIJI (image J) software] was performed by taking six different confocal images as samples for each strain at a time with the experimental repeat of at least thrice and represented with Mean ± SD. P-values for significant difference level were determined by performing student’s T-test (two tailed, paired). ***; p < 0.001. (TIF) [file pgen.1008395.s011.tif]

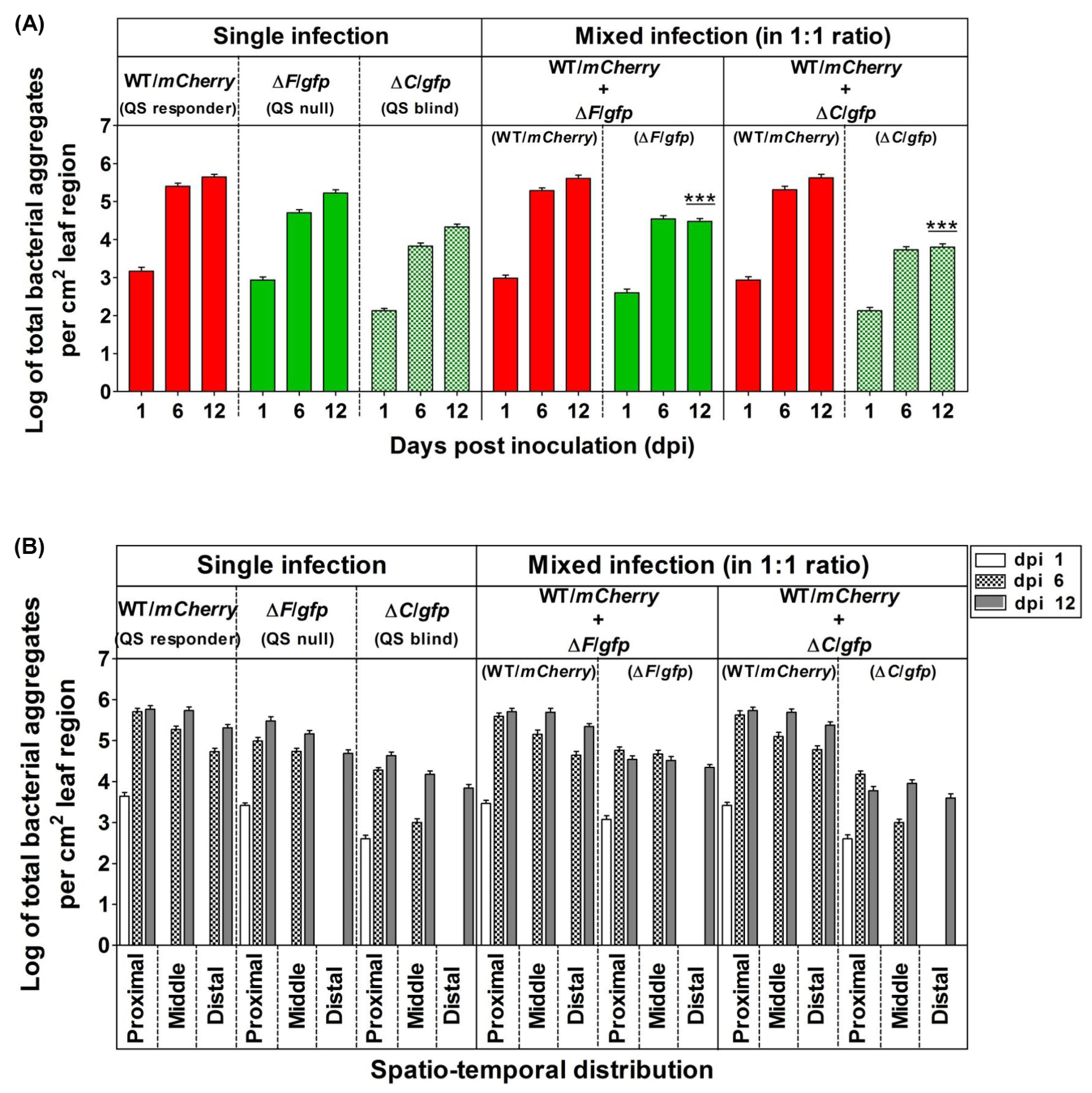

Supplement: S12 Fig — Cell aggregate formation patterns of QS responder (i.e. Xcc 8004/mCherry), QS null (i.e. Xcc ΔrpfF/gfp) and QS blind (i.e. Xcc ΔrpfC/gfp) cells for single as well as mixed infections in the in planta competition assay within clip inoculated cabbage leaves under a CLSM upto dpi 12. (A) Average no. of bacterial aggregates per 1 cm2 leaf regions, and (B) Spatio-temporal distribution of no. of bacterial aggregates per 1 cm2 proximal, middle and distal leaf regions respectively, for single and mixed infections on dpi 1, 6 and 12. WT; wild-type Xcc 8004, ΔF; Xcc ΔrpfF, and ΔC; Xcc ΔrpfC. On specified sampling dpi, multiple Z-stalks were acquired under a CLSM for each sample under green and red fluorescence along with DIC channel, maintaining 0.5 μm gap between two successive Z-planes. Bacterial aggregate size as well as no. were analysed by considering the X,Y and Z planes for each aggregate of a Z-stalk, where the bacterial aggregates present in all the Z-planes were counted manually and summed up to calculate their total no. in that region at a time. Total bacterial aggregate no. observed was normalized and the values are expressed per cm2 leaf region. The no. of bacterial aggregates for each infection was determined by combining the analysed data for five sites per inoculated leaf, six leaves on each sampling day with experimental repeats for thrice. The characteristics of the total region of the leaf observed at each sampling time were slightly different. Data analysis [using FIJI (image J) software] was performed by taking six different confocal images as samples for each strain at a time with the experimental repeat of at least thrice and represented with Mean ± SD. P-values for significant difference level were determined by performing student’s T-test (two tailed, paired). ***; p < 0.001. (TIF) [file pgen.1008395.s012.tif]

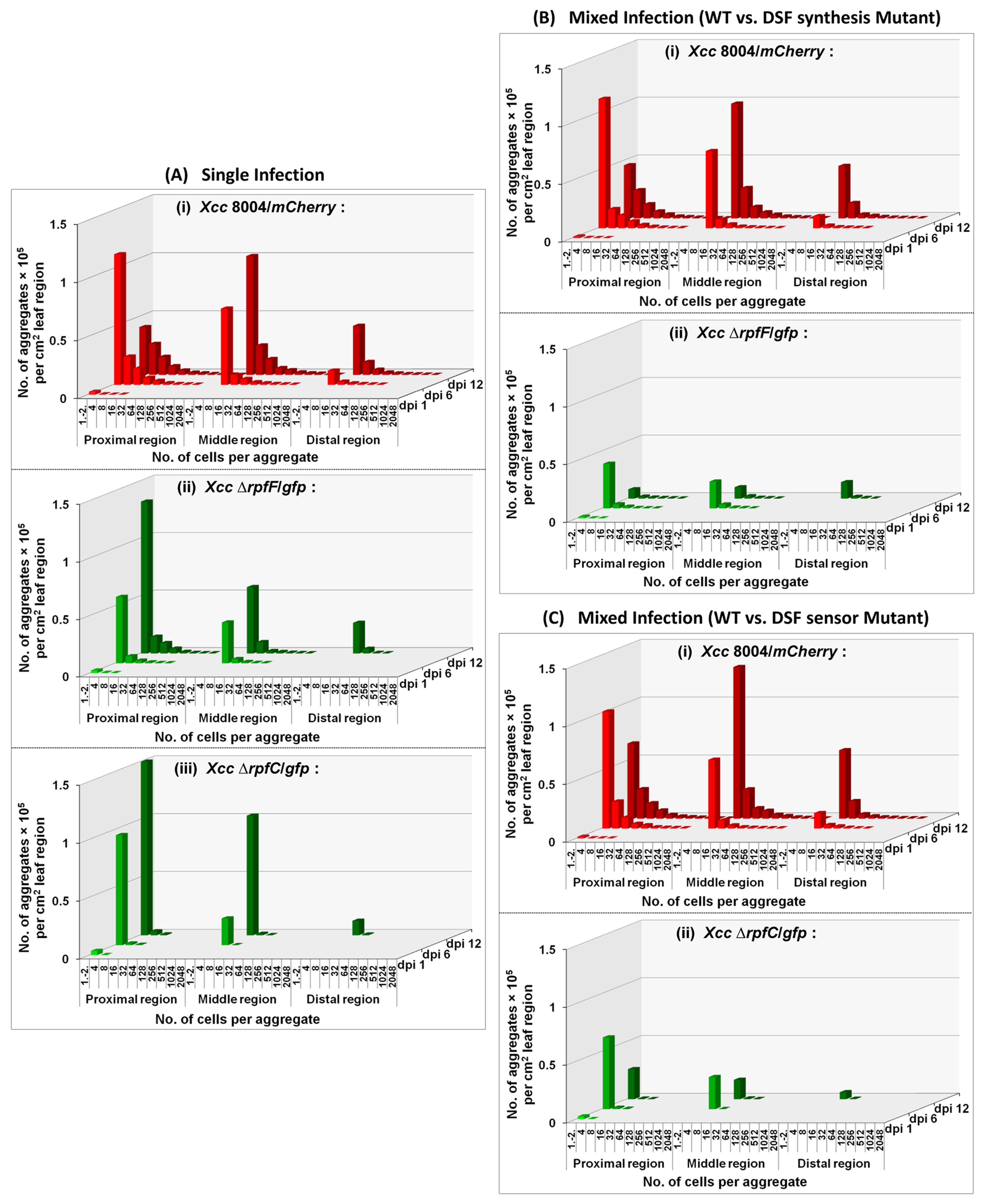

Supplement: S13 Fig — Frequency distribution of bacterial aggregates of Xcc 8004/mCherry (WT), Xcc ΔrpfF/gfp (DSF synthesis mutant) and Xcc ΔrpfC/gfp (DSF sensor mutant) bioreporter populations within inoculated cabbage leaves on dpi 1, 6 and 12 for individual as well as mixed (co-inoculated with 1:1 ratios, from ~ 107 cells ml-1 2o culture) infections. (A) Single Infection studies; with (i) Xcc 8004/mCherry (WT), (ii) Xcc ΔrpfF/gfp (DSF synthesis mutant) and (iii) Xcc ΔrpfC/gfp (DSF sensor mutant), (B) Mixed Infection studies; with (i) Xcc 8004/mCherry (WT) vs. (ii) Xcc ΔrpfF/gfp (DSF synthesis mutant), and (C) Mixed Infection studies; with (i) Xcc 8004/mCherry (WT) vs. (ii) Xcc ΔrpfC/gfp (DSF sensor mutant). On specific dpi, the bacterial aggregates were observed and their frequency distribution were calculated within proximal, middle and distal green regions to the infection site spanning approximately 1cm distance for each region. The total number of bacterial aggregates for each infection was determined for six leaves at each sampling time, and the data were combined. The characteristics of the total region of the leaf observed at each sampling time were slightly different. The number of cells per aggregate was estimated from the surface area and depth of each aggregate using FIJI (image J) software. The total number of aggregates observed was normalized; values are expressed per cm2 leaf region. (TIF) [file pgen.1008395.s013.tif]
